# BdlA, DipA and Induced Dispersion Contribute to Acute Virulence and Chronic Persistence of *Pseudomonas aeruginosa*

**DOI:** 10.1371/journal.ppat.1004168

**Published:** 2014-06-05

**Authors:** Yi Li, Olga E. Petrova, Shengchang Su, Gee W. Lau, Warunya Panmanee, Renuka Na, Daniel J. Hassett, David G. Davies, Karin Sauer

**Affiliations:** 1 Department of Biological Sciences, Binghamton University, Binghamton, New York, United States of America; 2 Department of Molecular Genetics, Biochemistry and Microbiology, University of Cincinnati College of Medicine, Cincinnati, Ohio, United States of America; 3 College of Veterinary Medicine, Urbana, Illinois, United States of America; University of Washington, United States of America

## Abstract

The human pathogen *Pseudomonas aeruginosa* is capable of causing both acute and chronic infections. Differences in virulence are attributable to the mode of growth: bacteria growing planktonically cause acute infections, while bacteria growing in matrix-enclosed aggregates known as biofilms are associated with chronic, persistent infections. While the contribution of the planktonic and biofilm modes of growth to virulence is now widely accepted, little is known about the role of dispersion in virulence, the active process by which biofilm bacteria switch back to the planktonic mode of growth. Here, we demonstrate that *P. aeruginosa* dispersed cells display a virulence phenotype distinct from those of planktonic and biofilm cells. While the highest activity of cytotoxic and degradative enzymes capable of breaking down polymeric matrix components was detected in supernatants of planktonic cells, the enzymatic activity of dispersed cell supernatants was similar to that of biofilm supernatants. Supernatants of non-dispersing Δ*bdlA* biofilms were characterized by a lack of many of the degradative activities. Expression of genes contributing to the virulence of *P. aeruginosa* was nearly 30-fold reduced in biofilm cells relative to planktonic cells. Gene expression analysis indicated dispersed cells, while dispersing from a biofilm and returning to the single cell lifestyle, to be distinct from both biofilm and planktonic cells, with virulence transcript levels being reduced up to 150-fold compared to planktonic cells. In contrast, virulence gene transcript levels were significantly increased in non-dispersing Δ*bdlA* and Δ*dipA* biofilms compared to wild-type planktonic cells. Despite this, *bdlA* and *dipA* inactivation, resulting in an inability to disperse *in vitro*, correlated with reduced pathogenicity and competitiveness in cross-phylum acute virulence models. In contrast, *bdlA* inactivation rendered *P. aeruginosa* more persistent upon chronic colonization of the murine lung, overall indicating that dispersion may contribute to both acute and chronic infections.

## Author Summary

Pathogenic bacteria, including the human pathogen *Pseudomonas aeruginosa*, can cause acute and chronic infections. The difference in these infection modes can be explained by how bacteria grow. Acute infections occur when individual bacteria rapidly replicate, produce high levels of virulence factors, and disseminate from the nidus of infection. Chronic infections occur when bacteria adhere to tissue or implanted medical devices and form multi-cellular, matrix-encased aggregates known as biofilms. The acute-to-chronic infection switch occurs when bacteria transition from planktonic to biofilm growth. However, the contribution of dispersion, the process by which bacteria leave a biofilm to return to planktonic growth, remains unclear. Here, we demonstrate that, while having left a biofilm, dispersed cells are distinct from planktonic cells with respect to gene expression, release of matrix-degrading enzymes, and pathogenicity. We found that a mutant impaired in nutrient-induced dispersion, while enhancing chronic infections, is impaired in mounting acute infections in both plant and mouse hosts. Overall, this work establishes that dispersed cells have a unique virulence phenotype, with nutrient-induced dispersion not only serving as an integral part of both acute and chronic infections but also as a potential mechanism of infection control.

## Introduction

*Pseudomonas aeruginosa* is a ubiquitous, Gram-negative opportunistic bacterial pathogen, well known for its remarkable ability to replicate and survive in diverse environments, as well as for causing a variety of acute and chronic human infections. Acute infections are characterized by rapid pathogenic progression, high-level toxin production, and often tissue damage. Chronic infections are characterized by colonization, prolonged persistence, evasion of the host's immune response and tolerance and often resistance to multiple therapeutic agents. The capacity to cause either acute or chronic infections depends to a large extent on the ability of *P. aeruginosa* to transit from growing planktonically (free living state) to surface-attached communities known as biofilm [1], [2], [3], [4], [5]. While planktonic infections are frequently associated with high virulence and fast growth as observed in sepsis or bacteremia [6], the development of biofilms is considered to be the root cause of chronic infections [1], [2], [3], [7], [8], [9]. Biofilms are composed of microorganisms attached to a solid surface and encased in a hydrated polymeric matrix composed of polysaccharides, protein and DNA. Biofilms form when bacteria adhere to surfaces in moist environments. For example, *P. aeruginosa* forms what are termed Mode II biofilms [10] within the airways of individuals with cystic fibrosis (CF), with this process playing an important role in CF-associated chronic infections and acting as one of the primary causes of mortality in CF patients [11], [12], [13], [14]. In addition, *P. aeruginosa* causes a variety of chronic infections in immunocompromised individuals or those suffering from wounds, burns, urinary tract infections, or corneal injury [7], [15], [16], [17], [18], [19].

The transition from a planktonic to surface-attached lifestyle is highly regulated. The two-component regulatory systems (TCS) including the RetS/GacS/GacA/rsmZ signal transduction pathway, as well as the TCS SagS and BfiSR are required for *P. aeruginosa* to transition to the surface associated mode of growth and to progress from initial attachment to the formation of mature biofilms [1], [2], [3], [4], [5], [20]. Additional regulatory systems required for the formation of mature, three-dimensional biofilms include the TCS BfmSR, MifSR, and SadARS [8], [21], [22]. However, these regulatory systems not only regulate the motile-sessile switch but also the growth-mode dependent expression of virulence factors. For example, genome-wide transcriptional profiling suggested that RetS is required for expression of the Type III secretion system (T3SS) genes (*psc* and *pcr* operons), secreted effectors ExoS, ExoT and ExoY [1], [23], [24], and other virulence factors, and for repression of genes responsible for exopolysaccharide components of the *P. aeruginosa* biofilm matrix [1]. Conversely, inactivation of *retS* correlated with hyper-adhesion to mammalian cells but loss of cytotoxicity and attenuated virulence in an acute pneumonia model. In addition to RetS and GacS/GacA/rsmZ, regulation of T3SS gene expression also requires SadARS [8]. Considering the number of regulatory systems affecting T3SS expression, it is not surprising that T3SS-mediated cytotoxicity is considered a key virulence mechanism of *P. aeruginosa*, allowing for the contact-dependent translocation of pathogenicity factors into eukaryotic host cells, with T3SS inactivation attenuating *P. aeruginosa* virulence in a murine acute infection model [20], [25], [26], [27], [28]. Additional known virulence factors produced by *P. aeruginosa* include hydrogen cyanide, elastase, phenazines, and rhamnolipids. Hydrogen cyanide is believed to be the primary toxic factor secreted by *P. aeruginosa* that is responsible for killing *Caenorhabditis elegans* [29], and has been detected at elevated levels in the infected lungs of patients suffering from CF [30]. Chitinase is expressed at high cell densities and in biofilms and was shown to be able to bind and degrade colloidal chitin [31], while elastase has been shown to cause localized tissue damage [32]. The redox-active pigments phenazines can generate an oxidative stress on the host and are required for “fast killing” of *C. elegans* [33]. Rhamnolipids interfere with the internalization of attached particles, reducing the level of phagosome-lysosome fusion of internalized targets within macrophages, and inhibiting the response of alveolar macrophages [34], [35].

While the clinical relevance of planktonic and biofilm cells in acute and chronic infections, respectively, has been well established, little is known about the contribution of biofilm dispersion to the virulence phenotype of *P. aeruginosa* and infections. Dispersion is the last yet a very important step in the development of biofilms that allows bacteria to successfully return from the biofilm to the planktonic growth state to spawn novel communities in new locales [36], [37]. Biofilm dispersion can be induced by exposure to matrix-degrading enzymes and surface protein releasing factors [38], [39], [40]. In *P. putida* and *P. fluorescens*, the large adhesive outer-membrane protein LapA mediates attachment to surfaces and to matrix components [41], [42], [43]. Gjermansen et al. [41] demonstrated that in *P. putida*, release of LapA from the cell surface results in biofilm dispersal and is mediated through the activity of the periplasmic protease LapG. Additional mechanisms linked to dispersion include cell death, with filamentous phage Pf1-mediated cell lysis serving as an important mechanism of differentiation inside microcolonies that facilitates dispersal of a subpopulation of surviving cells [44]. The process of biofilm dispersion in various organisms including *P. aeruginosa, P. putida* and *Schewanella oneidensis* is furthermore induced upon sensing a myriad of environmental cues such as variation in oxygen or carbon substrate concentration and sensing the signaling molecule *cis*-2-decenoic acid [36], [38], [45], [46], [47], [48], [49], [50]. Oxidative or nitrosative stress, induced upon exposure to exogenous or endogenous nitric oxide (NO), has been linked to biofilm dispersion. A role of oxidative or nitrosative stress was further supported by a *P. aeruginosa* mutant lacking the only enzyme capable of generating metabolic NO through anaerobic respiration (nitrite reductase, Δ*nirS*) not dispersing [51].

Biofilm dispersion has also been linked to the modulation of the intracellular signaling molecule cyclic di-GMP (c-di-GMP), high levels of which promote sessile growth, while low levels correlate with planktonic existence [52], [53], [54]. Levels of c-di-GMP are enzymatically modulated by diguanylate cyclases (DCG), proteins containing a GGDEF domain, and phosphodiesterases (PDE) harboring either an EAL or HD-GYP domain [52]. In *P. aeruginosa*, dispersion upon exposure to NO and elevated nutrient concentrations has been linked to the reduction of the cellular c-di-GMP levels, requiring the phosphodiesterases DipA and RbdA [46], [55], [56], [57]. In addition, the membrane-bound phosphodiesterase NbdA was found to be specific to the dispersion response following exposure to NO [48]. The chemotaxis transducer protein BdlA (Biofilm dispersion locus A) appears to play a central role in the dispersion response by *P. aeruginosa* biofilms, as inactivation of *bdlA* impaired dispersion by *P. aeruginosa* biofilms in response to various nutrients, NO, ammonium chloride, and heavy metals [56], [57]. However, BdlA does not directly contribute to the observed reduction of cellular c-di-GMP levels in dispersed cells as BdlA lacks domains required for c-di-GMP modulation. Instead, BdlA harbors two sensory Per Arnt Sim (PAS) domains and a chemoreceptor domain, TarH. The closest known BdlA homolog is the FAD-binding Aer, the redox potential sensor and aerotaxis transducer in *Escherichia coli*. Alanine replacement mutagenesis of BdlA-PAS domain residues D14A, N23A, W60A, I109A, W182A, that were previously demonstrated to be essential for aerotaxis in Aer, resulted in impaired dispersion, while alanine replacement mutagenesis of residue G31A resulted in the mutant strain transmitting a constant signal-on bias as it rendered *P. aeruginosa* biofilms hyper-dispersive [58]. The findings suggested BdlA to likely function as a sensory protein [58]. However, for BdlA to contribute to the dispersion response, it must first be activated via unusual, non-processive proteolytic cleavage at a ClpP-protease-like cleavage site located between the PAS sensory domains PASa and PASb within the BdlA protein [45]. Proteolysis of BdlA was stimulated by increased c-di-GMP levels present in biofilms, and dependent on the protease ClpP, the chaperone ClpD, and BdlA phosphorylation at tyrosine-238 [45]. Once activated, BdlA oligomerizes with the phosphodiesterases DipA and RbdA, thus forming a regulatory network that modulates the intracellular c-di-GMP pool to enable dispersion [57], [58].

Considering the link between the motile-sessile transition and the switch in *P. aeruginosa* virulence towards chronic infections [1], [2], we addressed herein whether induced dispersion, which enables the return to the planktonic mode of growth, is part of an inherent strategy of *P. aeruginosa* to initiate or contribute to either acute or chronic infections. To address this question, we analyzed virulence factor production and gene expression in cells exhibiting different growth modes by making use of dispersed cells obtained upon induction of dispersion in response to NO or changes in the nutrient glutamate concentration, as well as in a Δ*bdlA* mutant strain. This mutant was chosen as it is impaired in nutrient- and NO-induced biofilm dispersion [45], [56], [58] while not directly affecting c-di-GMP levels [56], [58]. This was done as intracellular signaling via c-di-GMP has been demonstrated to be an important contributor to virulence in multiple pathogens [59], [60], [61]. To assure, however, that the observed effects on virulence and pathogenicity are not specific to BdlA but instead linked to dispersion, we furthermore made use of mutants inactivated in *dipA* and *rbdA*.

## Results

### Biofilms prior to and post induction of dispersion release fewer degradative proteins than dispersed cells

**Figure 1:**
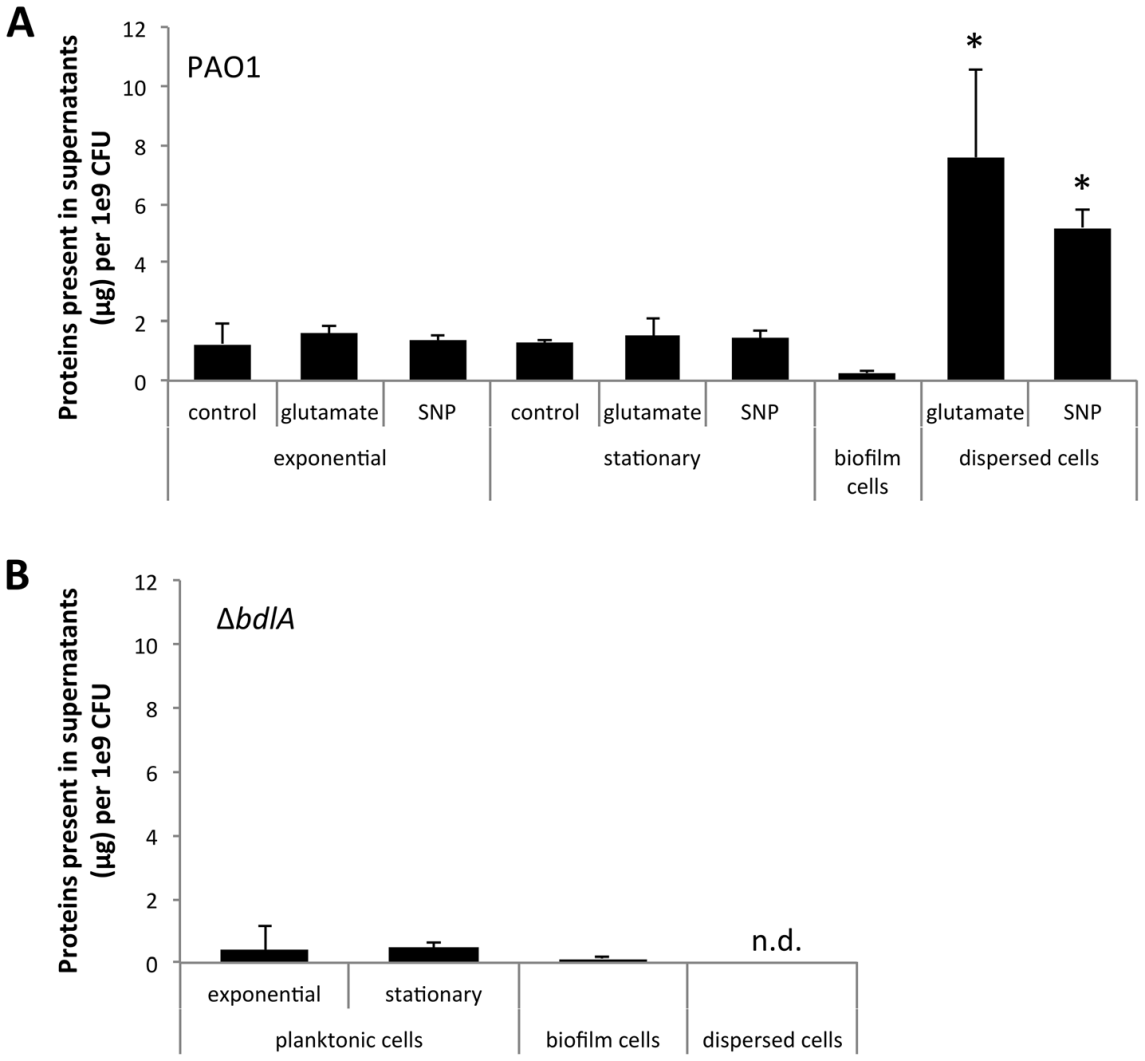
Dispersion of *P. aeruginosa* PAO1 biofilms correlates with increased release of proteins into the supernatant. Supernatants were obtained from (A) *P. aeruginosa* PAO1 and (B) Δ*bdlA* grown planktonically to exponential and stationary phase, as well as from biofilms and cells dispersed from the biofilm in response to exposure to glutamate (dispersed cells). Dispersed cells were obtained following dispersion in response to glutamate and SNP, which was used as a source of nitric oxide. Likewise, planktonic cells grown to exponential and stationary phase were exposed for 20 min to glutamate or SNP. Planktonic cells not treated with glutamate or nitric oxide are referred to as “control”. Experiments were carried out in triplicate. Error bars indicate standard deviation. The protein concentration of supernatants was determined using the same number of cells (1x10^9^ CFU/ml) regardless of growth conditions. n.d., not determined.

Several reports have described the process of dispersion to occur from within microcolonies, with dispersing bacteria observed to be motile, followed by them swimming away from the inner portions of the cell cluster through openings in the cluster and entering the bulk liquid [37], [47]. The *in vitro* observations suggested bacteria within microcolonies and enmeshed by extracellular polymeric substance (EPS) matrix composed of polysaccharides, extracellular DNA, lipids, and proteins (reviewed in [62], [63], [64]) to be able to liberate themselves in order to evacuate from biofilms. We, therefore, hypothesized that dispersion may correlate with the release of enzymes that assist in the degradation of the EPS matrix. To do so, we first determined the concentration of proteins present in culture supernatants produced by 1×10^9^ *P. aeruginosa* PAO1 bacteria grown planktonically, as biofilms, and following dispersion in response to nitric oxide (NO) or changes in the nutrient concentration, using the Bradford protein assay. NO was chosen as a dispersion inducing cue to mimic the endogenous production of or exogenous exposure to reactive oxygen intermediates to which *P. aeruginosa* is frequently exposed, both under anaerobic conditions prevalent in the CF lung environment and during infection [48], [51], [57]. Glutamate was used as a dispersion-inducing cue to mimic rapid environmental changes. Analysis of the culture supernatants of *P. aeruginosa* indicated the presence of supernatant proteins under all growth conditions tested (Fig. 1A). However, the highest amount of proteins was detected in supernatants of dispersed cells followed by supernatants from planktonic cells grown to exponential and stationary phase (Fig. 1A). The lowest concentration was routinely detected in biofilm supernatants (Fig. 1A). Overall, up to 10-times more protein was detected in supernatants of dispersed cells than those of planktonic cells (Fig. 1A). It is of interest to note that no difference in the concentration of supernatant proteins following dispersion in response to NO or changes in the nutrient concentration was noted, indicating protein release to be independent of dispersion inducing conditions (Fig. 1A). As dispersion was induced by NO or glutamate, we also analyzed culture supernatants of planktonic cells exposed to glutamate and NO. No difference in the concentration of protein detected in supernatants of planktonic cells and planktonic cells exposed to glutamate or NO was noted (Fig. 1A). Similar trends were observed upon the analysis of supernatants obtained from the hyper-virulent strain *P. aeruginosa* PA14 (Fig. S1).

**Figure 2:**
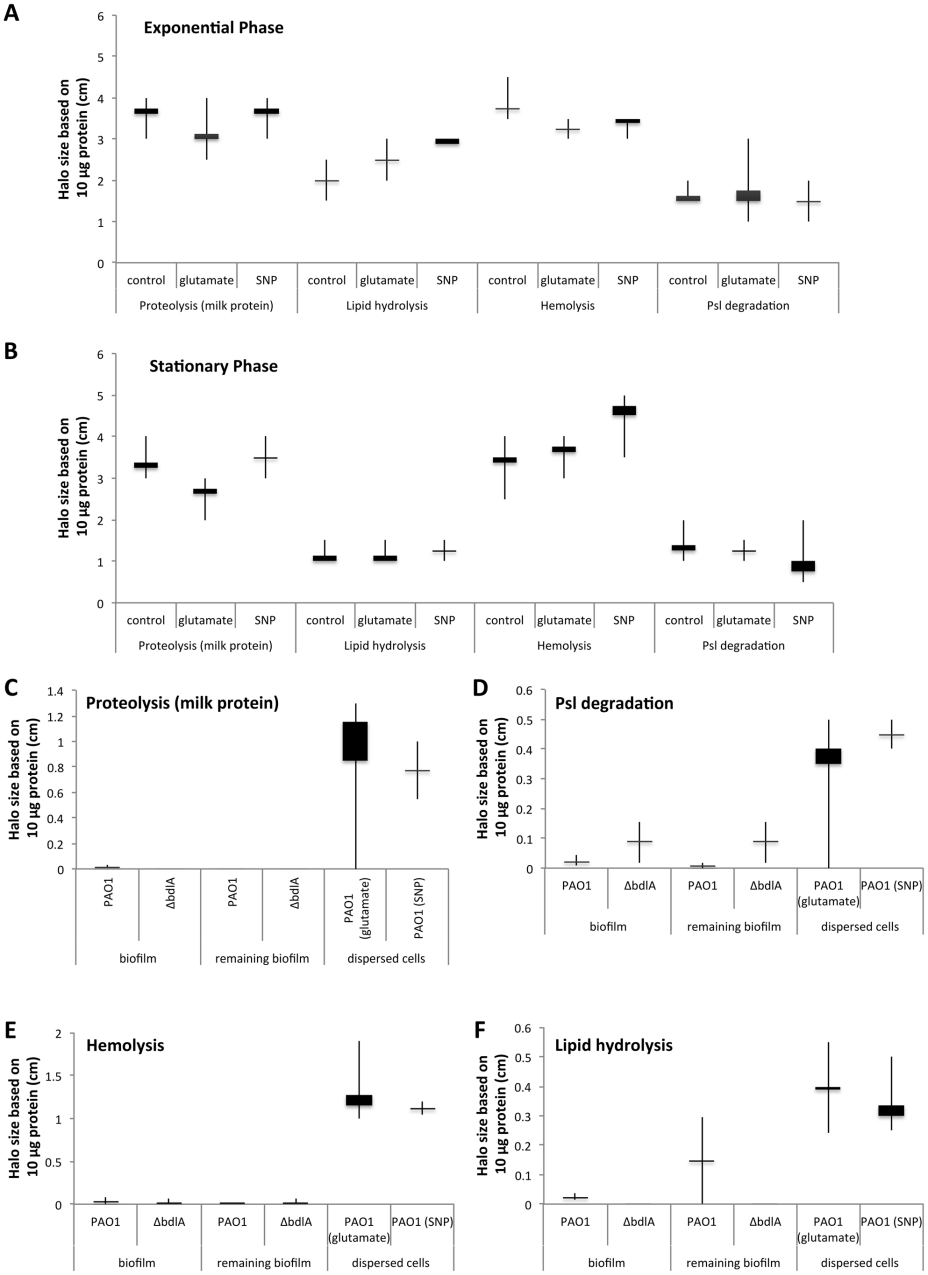
Detection of degradative activity in the extracellular proteome of *P. aeruginosa* PAO1 is growth mode dependent with *P. aeruginosa ΔbdlA* impaired in dispersion exhibiting lower degradative activity. Cytotoxic and degradative activities were determined using 10 µg of supernatant protein in 100 µl of sterile water, followed by measuring the zone of clearance 18 hr post-inoculation of the sterile protein solution into the wells of the respective agar plates. Degradative activity was determined using supernatants obtained from *P. aeruginosa* grown planktonically to exponential (A) and stationary phase (B). Supernatants of planktonic cells not treated with glutamate or nitric oxide are referred to as “control”. Additionally, supernatants of planktonic cells grown to exponential and stationary phase were exposed for 30 min to glutamate or SNP were used. (C–F) Degradative activities were furthermore determined in supernatants obtained from biofilms, and biofilms post-induction of dispersion with glutamate (remaining biofilm). Dispersed cells were obtained following dispersion in response to glutamate and SNP, which was used as a source of nitric oxide. (C) Proteolytic activity was detected using milk agar plates in supernatants obtained from biofilms, biofilms post-induction of dispersion, and dispersed cells. (D) Lipid hydrolysis was determined using tributyrin containing agar plates. (E) Hemolytic activity was detected using blood agar plates while (F) Psl degradation was detected on agar plates containing Psl extracted from a *P. aeruginosa* strain overexpressing Psl. Psl degradation was visualized as a zone of clearing following 24 hr incubation and staining the agar plate with iodine. Experiments were carried out at least in triplicate. Error bars indicate standard deviation.

Early infection and colonization have been suggested to require the production of a variety of cytotoxic and degradative proteins [1]. To determine whether proteins detected in the supernatants harbored cytotoxic or degradative activity capable of degrading polymeric tissue components or substances that may contribute to the release of bacterial cells from biofilms, we used agar plate-based assays, focusing on the detection of proteolysis, lipid hydrolysis, hemolysis, and Psl degradation. These degradative activities were chosen as they target components of the EPS matrix or are indicators of cytotoxicity. To better quantitate cytotoxic or degradative activities present in the various supernatants, a total of 10 µg of supernatant protein was used for each assay. Supernatants obtained from biofilms, remaining biofilms following nutrient-induced dispersion, dispersed cells following induction of dispersion in response to NO or changes in the medium glutamate concentration, and planktonic cells grown to exponential and stationary phase were used. In addition, supernatants obtained from planktonic cells exposed to NO and glutamate were used as controls. Supernatants obtained from *P. aeruginosa* PAO1 displayed proteolytic and Psl degradation activities as well as lipid hydrolysis and hemolysis, regardless of the mode of growth tested (Fig. 2). The highest activity with respect to proteolysis, lipid hydrolysis, hemolysis, and Psl degradation was detected in supernatants obtained from planktonic cells grown to exponential and stationary phase (Fig. 2A–B). Exposure of planktonic cells to NO or glutamate did not affect the overall activity present in supernatants (Fig. 2A–B). In contrast, with the exception of lipid hydrolysis, biofilms remaining attached following induction of dispersion demonstrated the least activity (Fig. 2). No significant difference in the cytotoxic and degradative activities was noted in supernatants of dispersed cells obtained following induction of dispersion in response to NO or changes in the nutrient glutamate concentration (Fig. 2C–F). Although supernatants of dispersed cells were characterized by levels approaching 10 times higher protein yield compared to planktonic and biofilm cells (Fig. 1A), the enzyme activity present in supernatants of dispersed cells was intermediate between that of biofilm and planktonic cells (Fig. 2). Overall, the cytotoxic and degradative activities present in 10 µg of supernatant proteins obtained from dispersed cells were, while elevated, more similar to those observed for biofilm supernatants than planktonic supernatants (Fig. 2). Similar results were observed when supernatants obtained from *P. aeruginosa* PA14 were used (Fig. S2). We furthermore determined DNA hydrolase activity in supernatants. The highest DNA hydrolase activity was found in supernatants of dispersed cells and biofilm cells remaining attached following induction of dispersion (Fig. S3).

### Supernatants obtained from the Δ*bdlA* mutant, impaired in biofilm dispersion, demonstrate reduced enzymatic activities

Our findings suggested that dispersed cells are distinct from planktonic and biofilm cells with respect to cytotoxic or degradative supernatant activities. To determine whether the ability to disperse correlated with the ability to degrade polymeric substances, we made use of a Δ*bdlA* mutant. Deletion of *bdlA* was previously demonstrated to render biofilm bacteria deficient in dispersion triggered by multiple environmental cues including various nutrients, NO, ammonium chloride, and heavy metals [45], [56], [58]. Compared to wild type bacteria, Δ*bdlA* grown planktonically and as biofilms released comparable amounts of proteins into the supernatant (Fig. 1B). Moreover, no difference in cytotoxic and degradative activity was noted for supernatants obtained from Δ*bdlA* mutant grown planktonically compared to that observed for the wild-type supernatants (not shown). Under biofilm growth conditions, however, very little enzymatic activity was detected in supernatants obtained from Δ*bdlA* biofilms and biofilms exposed to dispersion-inducing conditions (referred to as remaining biofilms, although no dispersion event occurred) compared to the wild type. In fact, no proteolysis and lipid hydrolysis was detected in supernatants from Δ*bdlA* biofilms and remaining biofilms, and hemolytic and Psl polysaccharide degradative activities were reduced compared to wild-type biofilms and remaining biofilms (Fig. 2C–F).

### Analysis of supernatant proteins of *P. aeruginosa* PAO1 and Δ*bdlA*

**Figure 3:**
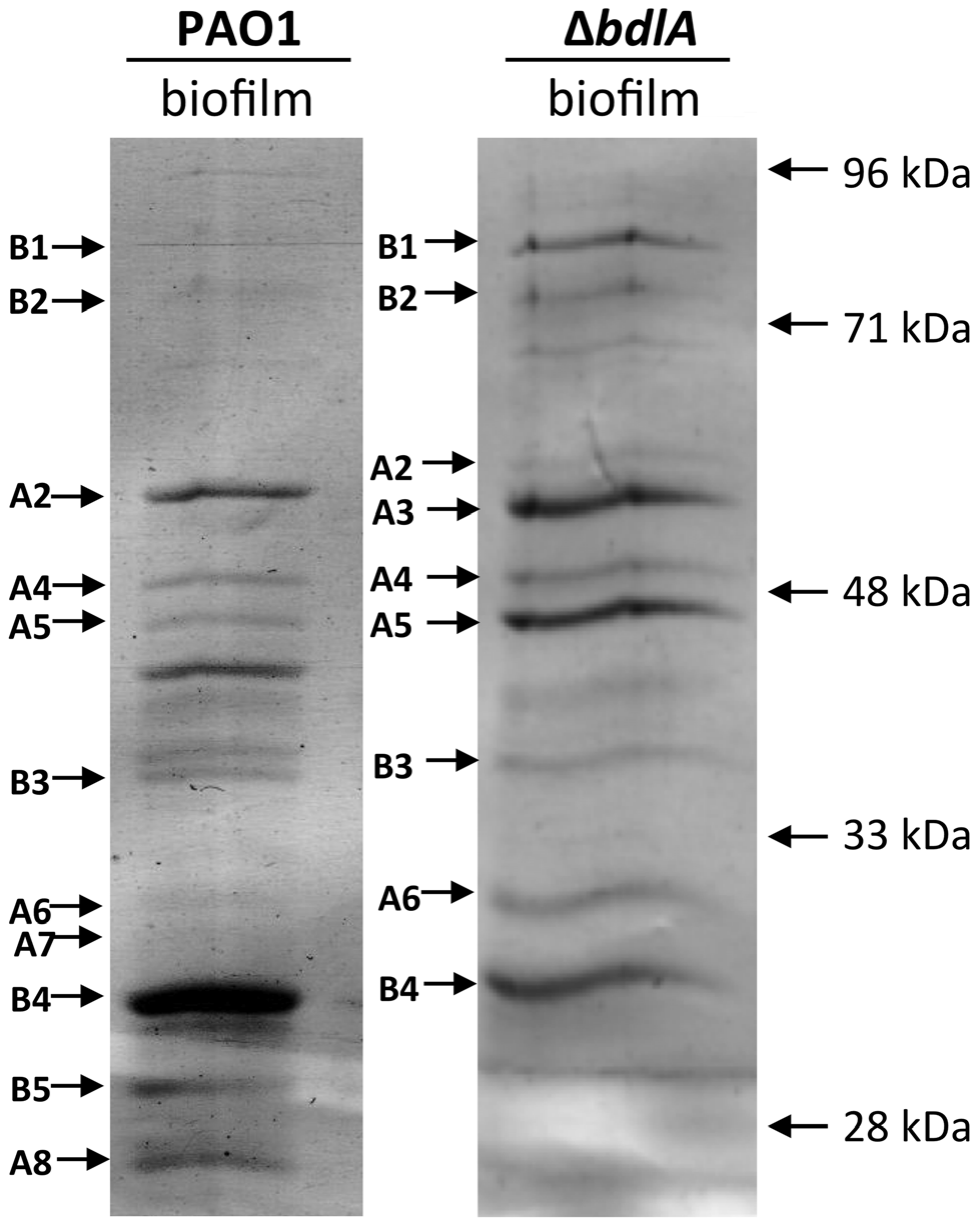
Analysis of proteins present in supernatants of *P.* aeruginosa PAO1 and *ΔbdlA* biofilms. A total of 10 µg supernatant protein obtained from *P. aeruginosa* PAO1 and *ΔbdlA* biofilms was loaded per lane. Protein bands indicated by a letter and arrow were selected and subsequently identified by LC-MS/MS (see also Table 1). Experiments were repeated in triplicate and a representative SDS-gel image is shown. Molecular masses are indicated on the right (in kDa).

Our findings not only suggested differences in *P. aeruginosa* PAO1 and Δ*bdlA* strains with respect to the release of degradative enzymes but, furthermore, that the inability to disperse likely corresponded with significantly reduced release or absence of degradative enzymes compared to wild-type biofilms. To further confirm differences in proteins released by the two strains, proteins present in supernatants obtained from biofilm cells were analyzed by SDS/PAGE, followed by subsequent protein identification by LC-MS/MS. As expected, marked differences in the extracellular proteins of wild-type and Δ*bdlA* mutant biofilms were noted (Fig. 3). However, the analysis also revealed the presence of a set of proteins common to the extracellular proteins of both wild type and the Δ*bdlA* mutant (Fig. 3). These included the outer membrane porins OprD, OprQ, and OprF as well as the flagellin component, FliC, the aminopeptidase PA2939, a component of the ABC transporter PA1342, and the sulfate binding protein precursor PA0283 (Fig. 3, Table 1). The latter two proteins were detected in a growth mode-dependent manner with PA1342 and PA0283 being absent in supernatants obtained from planktonic cells but detectable in supernatants obtained from biofilms (Fig. 3, Table 1). Differences in the extracellular proteome constituted the proteases elastase (LasB) and protease IV (PA3724). While both were detectable in supernatants of *P. aeruginosa* PAO1 under planktonic and biofilm growth conditions, the proteins were present at elevated levels in supernatants from *P. aeruginosa* PAO1 grown planktonically to stationary phase. In contrast, no protein bands of respective apparent mass were detected in culture supernatants of the Δ*bdlA* mutant (Fig. 3, Table 1). The only protease detected in the Δ*bdlA* mutant was the alkaline metalloproteinase, AprA (Fig. 3, Table 1). The finding of the Δ*bdlA* strain lacking proteases is consistent with the reduced proteolytic activity in this strain (Fig. 2C). No lipases or hemolysins were detected. Additional differences in the extracellular proteome were noted with respect to exotoxin A. Production of exotoxin A inhibits eukaryotic host cell protein synthesis by interacting with elongation factor-2, resulting in cell death [65]. While exotoxin A was detected in the supernatants of *P. aeruginosa* PAO1, its presence was limited to biofilms (Fig. 3, see black arrow “F2”, Table 1). In contrast, exotoxin A was detectable in supernatants of the Δ*bdlA* mutant regardless of growth conditions (Fig. 3, Table 1).

**Table 1:** Identification of proteins present in supernatants.

	Protein abundance				Protein ID	
Protein #	PAO1		Δ*bdlA*		PA locus	Description
	Plk.	Biofilm	Plk.	Biofilm		
A1	-	-	++	-	PA2452	Hypothetical protein
A2	++	+++	+	+	PA2939	Aminopeptidase
A3	-	-	+	+++	PA1429	AprA
A4	++	++	+	++	PA0958	OprD, outer membrane porin
A5	++	++	+	+++	PA2760	OprQ
A6	++	+	+	++	PA1777	OprF, structural outer membrane porin
A7	++	+	-	-	PA3724	LasB
A8	+++	++	-	-	PA4175	Protease IV
B1	-	+	+	++	PA2398	FpvA, ferripyoverdine receptor
B2	-	+	+	++	PA1148	Exotoxin A (ToxA)
B3	-	+	-	+	PA0283	sbp, sulfate-binding protein precursor
B4	-	+++	-	++	PA1342	binding protein component of ABC transporter
B5	-	++	-	-	PA2204	binding protein component of ABC transporter

### The virulence phenotype of nutrient-induced dispersed cells is distinct from that of planktonic cells

**Figure 4:**
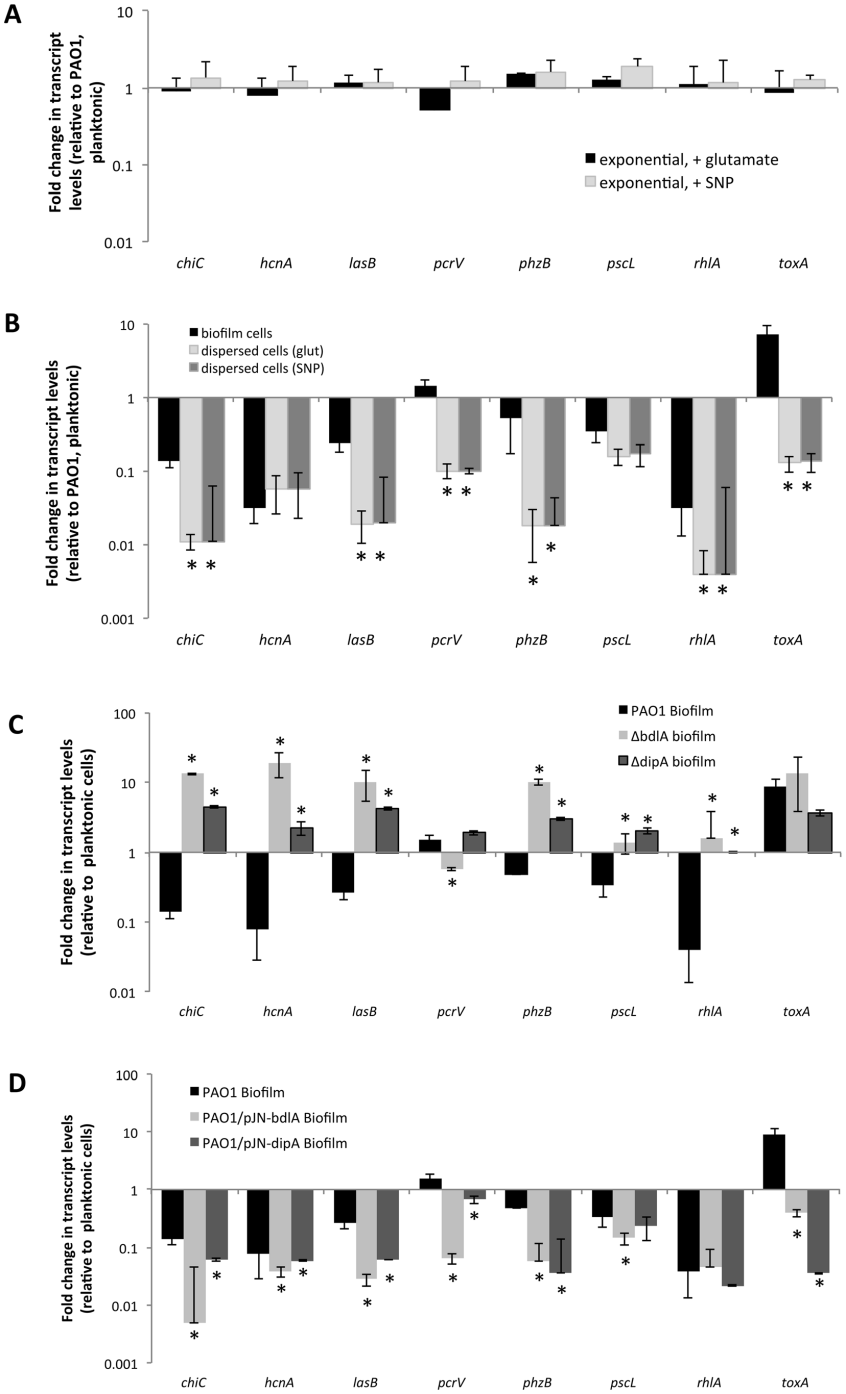
Expression of virulence genes in dispersed cells is distinct from the virulence gene expression profile of planktonic and biofilm cells. (A) Differential expression of selected virulence genes by *P. aeruginosa* PAO1 grown planktonically to exponential compared to expontial phase planktonic cells that were exposed for 30 min with 18 mM glutamate or 500 µM SNP, which was used as a source of nitric oxide [51]. (B) Differential expression of selected virulence genes by *P. aeruginosa* PAO1 grown as biofilms and dispersed cells compared to cells grown planktonically to exponential phase. Differential gene expression was determined by qRT-PCR. Dispersed cells were obtained following induction of dispersion by SNP (nitric oxide) or glutamate. *, significantly different from PAO1 grown as biofilms, P-value <0.01. (C) Differential expression of selected virulence genes by biofilm cells of *P. aeruginosa* PAO1 wild type and *ΔdipA* and *ΔbdlA* mutant strains compared to wild-type cells grown planktonically to exponential phase. *, significantly different from PAO1 grown as biofilms, P-value <0.01. (D) Differential expression of selected virulence genes by *P. aeruginosa* PAO1, PAO1/pJN-*bdlA*, and PAO1/pJN*-dipA* biofilms compared to wild-type cells grown planktonically to exponential phase. Differential gene expression was determined by qRT-PCR. Experiments were carried out at least in triplicate. *, significantly different from PAO1 grown as biofilms, P-value <0.01.

The difference in protease and exotoxin A abundance in the extracellular proteomes obtained from *P. aeruginosa* wild-type and Δ*bdlA* biofilms suggested a contribution of dispersion to the production of degradative and virulence factors. To further explore whether dispersion and the return to the planktonic, free-living mode of growth contributes to a switch in virulence gene expression, qRT-PCR was used to quantitatively determine the transcript levels of genes encoding several known virulence factors. In addition to exotoxin A, expression of the virulence factors hydrogen cyanide (*hcnA*), chitinase (*chiC*), elastase (*lasB*), pyocyanin (*phz* operon), genes encoding T3SS components (*pcrV* and *pscL*), and rhamnolipids (*rhlA*) [66], [67] was assessed. Considering that dispersed cells are exposed to NO or glutamate, we first determined whether expression of virulence genes is affected by the addition of NO or glutamate by analyzing the transcript abundance of virulence genes of interest in exponential phase planktonic cells and exponential phase planktonic cells exposed to glutamate and NO. No significant difference in transcript levels of the tested virulence genes was noted (Fig. 4A) indicating that under the conditions tested, exposure to NO or additional glutamate does not affect the expression of the virulence genes. We therefore compared transcript abundance of virulence genes in biofilms and dispersed cells to untreated exponential phase planktonic cells. Compared to planktonic cells, *P. aeruginosa* PAO1 grown as biofilms were characterized by significantly reduced expression of *chiC, lasB, rhlA, phzB*, *pscL*, and *hcnA*, but increased *pcrV* and *toxA* expression (Fig. 4B, Table S1). Increased *toxA* transcript levels in *P. aeruginosa* PAO1 biofilms are in agreement with the biofilm-specific detection of exotoxin A (Fig. 3). Dispersed cells were likewise characterized by reduced expression of virulence genes, regardless of whether NO or changes in the glutamate concentration were used to induce dispersion (Fig. 4B, Table S1). However, all virulence genes tested including *pcrV* and *toxA* were significantly reduced in dispersed cells compared to both planktonic or biofilm cells (Fig. 4B, Table S1). Similar changes in transcript levels were observed for dispersed *P. aeruginosa* PA14 cells compared to planktonic and biofilm cells (Fig. S4) and suggested that dispersion induced upon exposure to environmental cues to be more than merely a transitional episode of cells leaving the biofilm and adapting to the planktonic mode of growth.

Considering the similarity in the trend of virulence gene expression between biofilms and dispersed cells compared to planktonic cells, we hypothesized that dispersion may contribute to virulence gene expression and assessed this hypothesis by testing the dispersion-deficient Δ*bdlA* mutant strain grown as a biofilm. We anticipated finding transcript levels of virulence genes to be higher in biofilms impaired in dispersion compared to wild-type biofilms. qRT-PCR analysis revealed that transcript levels of *chiC, hcnA, lasB, phzB* were significantly increased in Δ*bdlA* biofilms relative to wild-type planktonic cells (and biofilm cells). In contrast, the transcript levels of *rhlA* and the T3SS genes *pscL* and *pcrV* were similar those observed for wild-type planktonic cells (but significantly increased compared to wild-type biofilm cells, Fig. 4C, Table S1). The only transcript present at similar levels in Δ*bdlA* biofilm and wild-type biofilm cells was *toxA*. The finding is in agreement with the supernatant protein analysis (Figs. 3, 4C, Table S1). It is of interest to note that similar results were obtained when virulence gene transcript levels of the dispersion-deficient Δ*dipA* mutant were determined (Fig. 4C).

In contrast, multi-copy expression of *bdlA*, which renders PAO1/pJN-*bdlA* biofilms hyper-dispersive [45], [58], resulted in significantly reduced expression of *chiC, lasB, rhlA, phzB*, *pscL, pcrV*, and *hcnA* compared to planktonic cells. Moreover, with the exception of *rhlA* and *toxA*, the transcript levels of *chiC, lasB, phzB*, *pscL, pcrV*, and *hcnA* detected in PAO1/pJN-*bdlA* biofilms were overall reduced compared to wild type biofilms (Fig. 4D, Table S1). Instead, the transcript levels of virulence genes in this hyper-dispersive biofilm were overall similar to those observed for dispersed cells (Fig. 4B-C, Table S1). A similar trend was observed upon overexpression of *dipA* in biofilms which has previously been demonstrated to also render *P. aeruginosa* biofilms hyper-dispersive [46]. With the exception of *pscl* and *rhlA*, the transcript levels of virulence genes detected in PAO1/pJN-*dipA* biofilms were significantly different relative to wild type biofilms (Fig. 4D). Our findings indicated non-dispersing and hyper-dispersing biofilms to express virulence genes in a manner distinct from biofilm and planktonic cells, with non-dispersing Δ*bdlA* and Δ*dipA* biofilms demonstrating increased expression of virulence genes compared to biofilm and planktonic cells while hyper-dispersing biofilms appeared to express virulence genes at reduced levels compared to planktonic and biofilm cells, with the expression profile overall being more similar to that of dispersed cells. Moreover, our findings further indicate that *toxA* expression is specific to the biofilm mode of growth.

### Inactivation of *bdlA* and impaired nutrient-induced dispersion capability attenuates the virulence of *P. aeruginosa* in an *Arabidopsis thaliana* infection model

**Figure 5:**
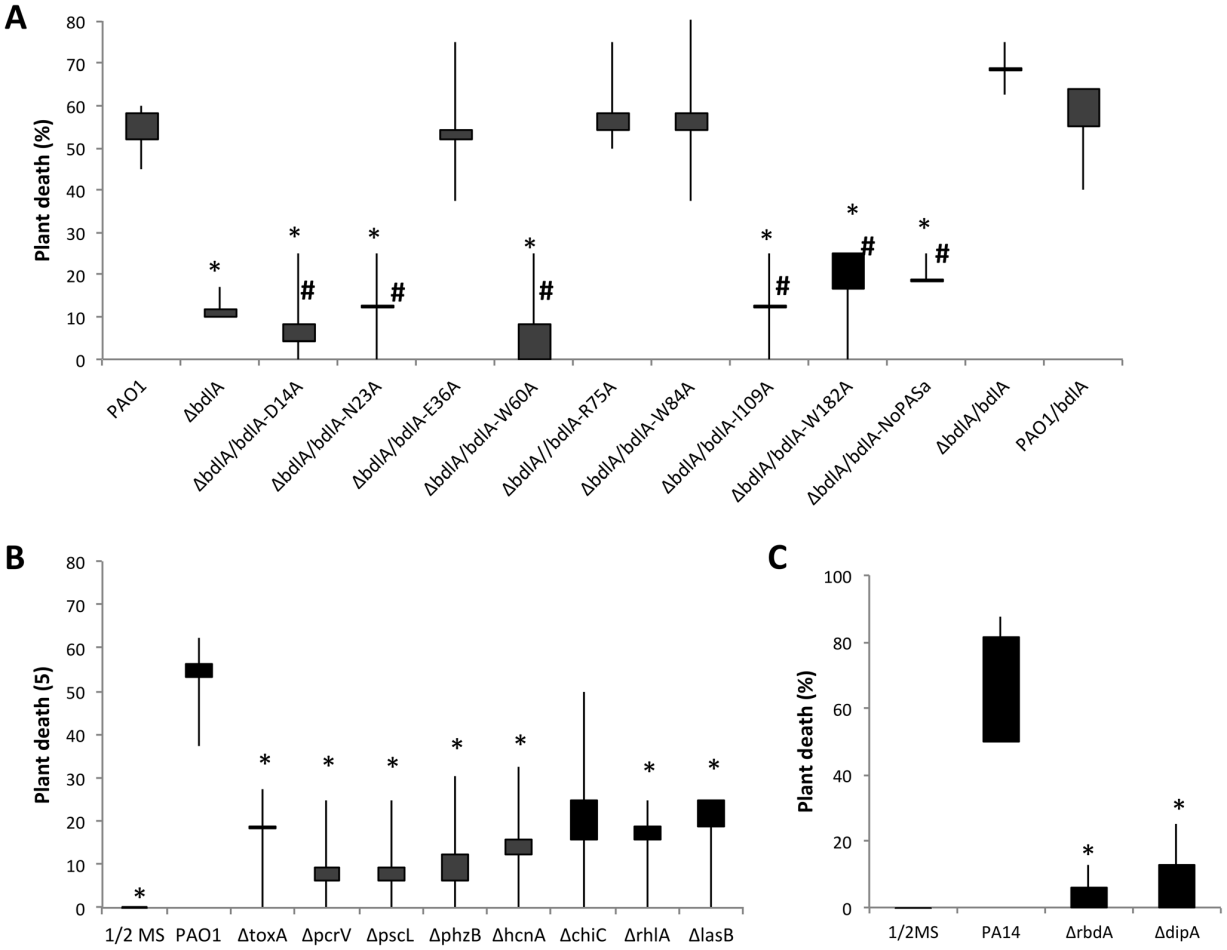
The non-dispersing Δ*bdlA* mutant and complemented Δ*bdlA* mutants impaired in biofilm dispersion are avirulent. (A) Death of *Arabidopsis thaliana* 7 days post infection with *P. aeruginosa* PAO1, the isogenic Δ*bdlA* mutant and *ΔbdlA* mutants complemented with *bdlA*, a truncated BdlA variant (NoPAS-*bdlA*) and BdlA variants harboring alanine substitutions in various amino acids. #, indicates complemented Δ*bdlA* strains impaired in nutrient-induced dispersion, see [58]. All other complemented Δ*bdlA* strains were not impaired in nutrient-induced dispersion. (B) Death of *Arabidopsis thaliana* 7 days post-infection with *P. aeruginosa* PAO1 and selected isogenic mutants. Bars indicate average and median plant death rates while vertical lines indicate the highest and lowest plant death rates observed. (C) Death of *Arabidopsis thaliana* 7 days post infection with *P. aeruginosa* PA14, and the isogenic Δ*dipA* and *ΔrbdA* mutants. Control plants inoculated with ½ MS salts alone showed no symptoms over the course of the experiments. Experiments were carried out in triplicate using 8 plants per strain per replicate. *, significantly different from PAO1, P-value <0.05.

Inactivation of *bdlA* correlated with increased expression of virulence factors but reduced release of cytotoxic and degradative enzymes relative to wild-type cells. In contrast, overexpression of *bdlA* resulted in significantly reduced transcript levels of virulence genes. We therefore asked whether *bdlA* inactivation affected the virulence phenotype of this mutant strain using an *A. thaliana* virulence model. This alternative nonvertebrate, plant host model was chosen as it has been previously demonstrated to result in the identification of bacterial virulence factors and to correlate with virulence outcomes obtained using vertebrate infection models such as the burned mouse pathogenicity model [68], [69]. Compared to the wild type, mutants lacking BdlA were less virulent. While more than 58% of *Arabidopsis* plants were killed by *P. aeruginosa* PAO1 within 7 days post infection (Fig. 5A) with the percent of dead plants rising to 70% following 9 days of infection (not shown), the isogenic Δ*bdlA* mutant was unable to establish infection within 7 days. Only 12% of all plants were killed 7 days post infection (Fig. 5A). In contrast, overexpression of *bdlA*, which was found to mimic a hyper-biofilm dispersion phenotype *in vitro* [45], [58], had no additional effect on *P. aeruginosa* virulence and plant mortality compared to the wild type (Fig. 5A).

To ensure that the factors encoding the tested virulence genes *chiC, lasB, rhlA, phzB*, *pscL*, *hcnA pcrV* and *toxA* contribute to the pathogenicity of *P. aeruginosa* in the *A. thaliana* virulence model, mutants inactivated in these virulence factors were tested. Inactivation of *exoA*, *hcnA*, *chiC*, *lasB*, *phz*, and *rhlA* and T3SS-coding genes *pcrV* and *pcsL* resulted in significantly reduced plant death compared to the wild type. While more than 55% of *Arabidopsis* plants were killed by *P. aeruginosa* PAO1 within 7 days post infection, infections with mutants inactivated in these virulence genes (with the exception of *chiC*) resulted in significantly reduced plant mortality, with less than 20% of all plants being killed over the same period of time (Fig. 5B). The findings confirmed a contribution of the respective virulence factors to *P. aeruginosa* pathogenicity. Considering, however, that the majority of these factors are up-regulated in Δ*bdlA* biofilms but reduced in PAO1/pJN-*bdlA* relative to wild-type biofilms, our findings further suggested that the ability to disperse, rather than the differential expression of a particular set of virulence factors, is contributing to the pathogenicity of *P. aeruginosa*.

To exclude the possibility that the effect on virulence was due to the absence of BdlA rather than linked to the ability to disperse, we made use of recently identified site-directed mutants of BdlA that were unable to restore the Δ*bdlA*-dispersion phenotype to wild-type levels [58]. These included alanine substitutions of the amino acids D14, N23, W60, I109, and W182 in BdlA. Δ*bdlA* mutants complemented with any of these BdlA variants were as avirulent as a Δ*bdlA* mutant (Fig. 5A). Similarly, complementation of Δ*bdlA* with a truncated BdlA lacking the N-terminal located PAS domain of BdlA, which was previously shown to not restore the Δ*bdlA* dispersion phenotype to wild-type levels [58], resulted in such a *bdlA* strain remaining avirulent (Fig. 5A). In contrast, alanine substitution in amino acid positions E36, R75, and W84 that did restore the biofilm-deficient phenotype of Δ*bdlA* to wild-type levels [58], rendered the complemented *bdlA* strain as virulent as wild-type bacteria (Fig. 5A). Similarly, a Δ*bdlA* mutant complemented with intact *bdlA* was used as positive control and found to be as virulent as the wild type (Fig. 5A). To furthermore support the link between dispersion and virulence, we additionally tested the mutant strains Δ*dipA* and Δ*rbdA*, that have been previously demonstrated to be impaired in dispersion of *P. aeruginosa* biofilms in response to NO and glutamate [46], [55]. Compared to the wild type, mutants lacking DipA and RbdA were significantly less virulent (Fig. 5C). While up to 80% of *Arabidopsis* plants were killed by the parental *P. aeruginosa* strain within 7 days post infection (Fig. 5C), the isogenic Δ*rbdA* and Δ*dipA* mutants were unable to establish infections. Overall, less than 10% of all plants were killed 7 days post infection (Fig. 5C). Our findings strongly indicate that the process of dispersion is a major contributor to *P. aeruginosa* pathogenicity, as strains impaired in dispersion are avirulent, while strains capable of the dispersion response demonstrated wild-type virulence levels.

### Inactivation of *bdlA* reduces competitiveness and attenuates the virulence of *P. aeruginosa* in a murine acute pneumonia model

**Figure 6:**
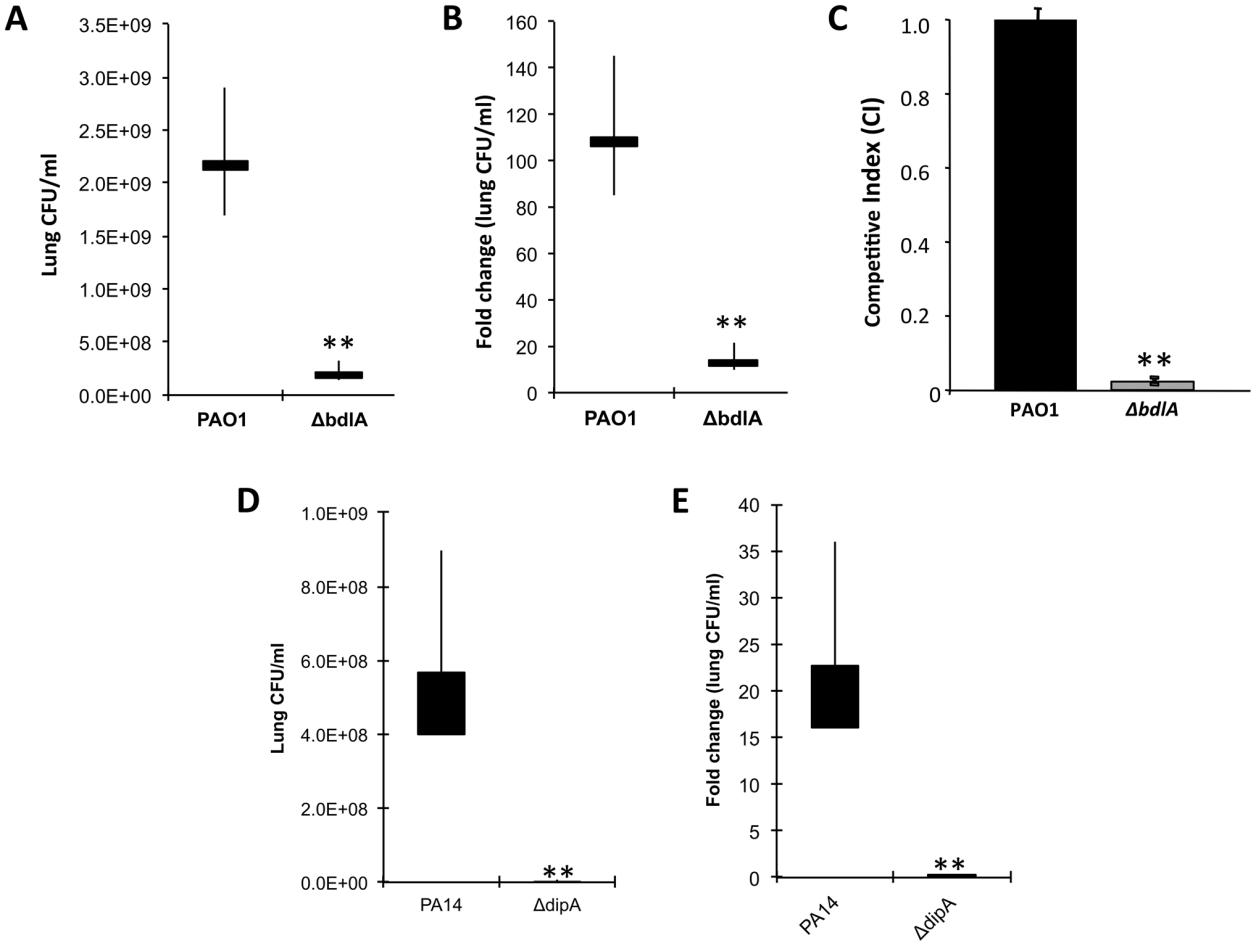
The non-dispersing *P. aeruginosa ΔbdlA* and *ΔdipA* mutant strains are less virulent and competitive compared to *P. aeruginosa* wild type as determined using an acute murine pneumonia infection model. CD1 mice were inoculated (intranasal) with 2×10^7^ CFU of *Pseudomonas* strains; lungs were harvested 24 h post-inoculation and CFU was determined. Values presented are average, min, max, mean. (A) Bacterial burden in the lung and (B) fold change in lung CFU/ml 24 hr post-infection compared to initial inoculum by *P. aeruginosa* PAO1 and the isogenic Δ*bdlA* mutant. Bars indicate average and median lung CFU/ml while vertical lines indicate the highest and lowest lung CFU/ml observed. (C) Competitive index was determined using mixed infection. *bdlA* is less competitive in an acute murine pneumonia infection model when co-inoculated with *P. aeruginosa* PAO1. Mice were inoculated intranasally with 1×10^7^ cells (1∶1 ratio) of wild type PA01 and isogenic Δ*bdlA* mutant. Error bars indicate standard deviation. (D) Bacterial burden in the lung and (E) fold change in lung CFU/ml 24 hr post-infection compared to initial inoculum by *P. aeruginosa* PA14 and the isogenic Δ*dipA* mutant. Bars indicate average and median lung CFU/ml while vertical lines indicate the highest and lowest lung CFU/ml observed. A total of 5 mice were used in per study. The values were tested by means of a Fisher test. **, significantly different from wild type (PAO1 or PA14), P-value <0.001.

To determine whether reduced virulence in a plant model of infection correlates with reduced acute infection and attenuation of virulence *in vivo*, we next examined the ability of the Δ*bdlA* mutant, which is impaired in dispersion *in vitro,* to colonize in an acute infection model, using a murine model of acute pneumonia. Wild-type *P. aeruginosa* is able to colonize the lungs of an infected mouse and grow about 100-fold in the course of 24 hr (Fig. 6A–B). The isogenic Δ*bdlA* mutant, in comparison, was unable to establish infection; bacterial load was reduced by 10-fold as compared to infection with the wild-type parental strain (Fig. 6A–B). Additionally, the *P. aeruginosa ΔbdlA* mutant was unable to establish systemic infections and no bacteria were recovered from the liver or spleen (data not shown).

Competitive mixed infection assays have been widely used to assess the fitness of individual *P. aeruginosa* mutants versus their parental strains during *in vivo* infection [70], [71]. Wild-type and Δ*bdlA* mutant bacteria were used to infect adult CD-1 mice (in groups of five) intranasally with 1×10^7^ cells (1∶1 ratio). Following 16 hr, infected lungs were recovered for bacterial load determinations. Remarkably, the Δ*bdlA* mutant was only 2% as (or 45-fold less) competitive as its parental strain PAO1 (Fig. 6C). To further ensure that the effect on virulence was linked to the ability to disperse, we also examined the ability of the Δ*dipA* mutant to establish an acute infection. Compared to the parental strain, the isogenic Δ*dipA* mutant, in comparison, was unable to establish infection, as apparent by the bacterial load being reduced by 80-fold compared to the wild-type strain (Fig. 6D). Moreover, while the bacterial load of wild-type bacteria increased about 20-fold in the course of 24 hr compared to the initial inoculum titer, the Δ*dipA* mutant load was reduced 4-5-fold (Fig. 6). The results suggested BdlA and DipA, and thus, likely the process of dispersion to play an important role in virulence of *P. aeruginosa* during acute infection of mouse airways.

### Inactivation of *bdlA* contributes to the persistence of *P. aeruginosa* in a murine model of chronic pneumonia

To determine the contribution of *bdlA* and dispersion to the ability of *P. aeruginosa* to persist at the site of infection *in vivo*, we next tested this mutant in a murine model of chronic pneumonia. Based on the Δ*bdlA* dispersion-deficient phenotype *in vitro*, we expected the *P. aeruginosa* Δ*bdlA* mutant to have an advantage in establishing biofilms and/or persistent infection compared to the wild type, due to its reduced virulence phenotype and the reduced number of cells released from the biofilm. We made use of a chronic infection model established by Cash et al. [72] except that we made use of a murine rather than a rat model. The model is based on the intratracheal administration of agarose beads impregnated with *P. aeruginosa*. This model was chosen because it allows the study of chronic infection, marked by the formation of persistent bacterial biofilm populations at the site of infection that can persist for up to 6 months and mimic biofilm-related infections [70], [71], [73], [74], [75].

**Figure 7:**
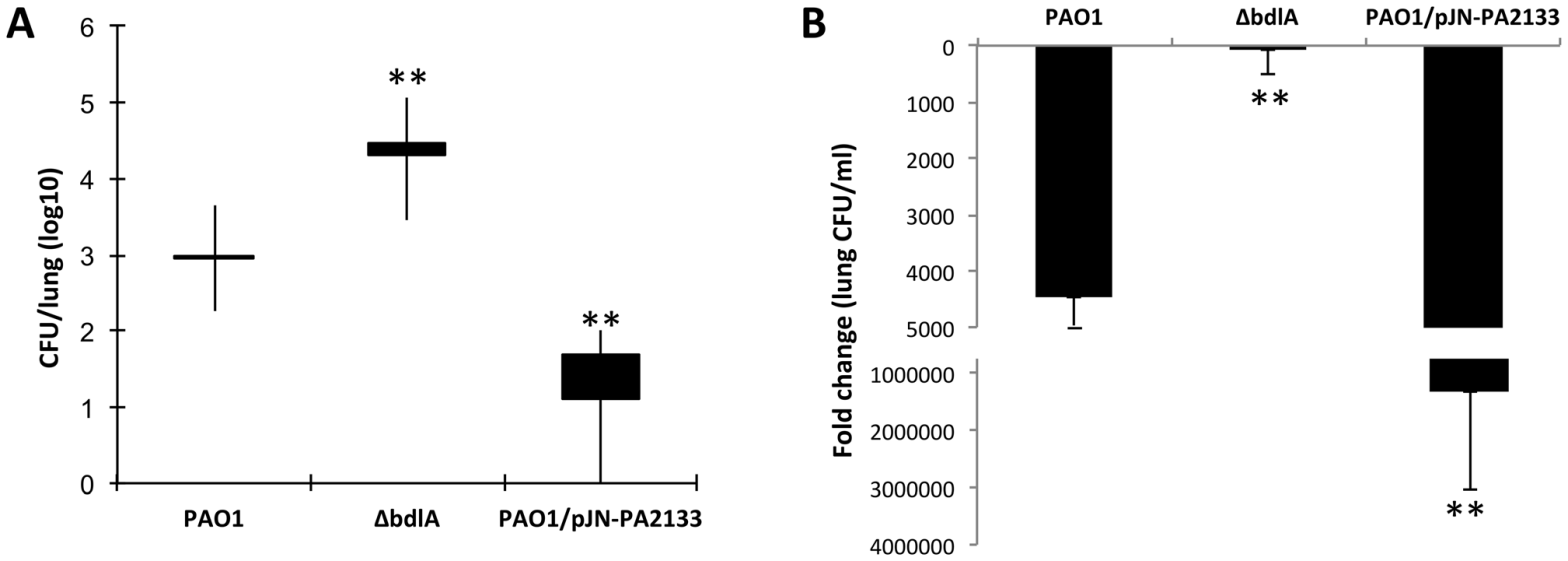
Impaired dispersion correlates with increased persistence as determined using a chronic murine pneumonia infection model. CD1 mice were inoculated intratracheal through oral lavage using feeding needle with 1.2×10^6^ CFU of *Pseudomonas* strains. Lungs were harvested 14 days post-inoculation and CFU was determined. A total of 10 mice were used in per study. (A) Bacterial burden in the lung. Values presented are average, min, max, mean lung CFU/ml. (B) Fold change in lung CFU/ml 14 days post-infection compared to initial inoculum. Error bars indicate standard deviation. The values were tested by means of a Fisher test. **, significantly different from PAO1, P-value <0.001.

Following 14 days of infection, the bacterial load in the lungs of mice infected with Δ*bdlA* was significantly higher (1.5-log increase) than that of *P. aeruginosa* PAO1 (Fig. 7). Moreover, compared to the initial inoculum (1.2×10^6^ CFU), a less than a 100-fold reduction was noted for Δ*bdlA* while a more than 4000-fold reduction was detected between the initial inoculum and the bacterial burden 14 days post infection for the wild type. To ensure that the bacterial load in the lungs coincided with biofilm formation, we also tested *P. aeruginosa* overexpressing PA2133. PA2133 encodes a phosphodiesterase, and PAO1 strain overexpressing the PA2133 was previously shown by Hickman et al. [76] to be significantly impaired in biofilm formation *in vitro*, even after 72 h of incubation. Reduced bacterial burden correlated with reduced or impaired biofilm formation as indicated by the reduced detection of PAO1/pJN-PA2133 bacterial cells in the lung. Compared to PAO1/pJN-PA2133, the bacterial burden by *P. aeruginosa* PAO1 was increased by 1.5 logs, and Δ*bdlA* showed a 4 log increase (Fig. 7). Decreased burden correlated with a significant reduction in bacterial load compared to the original inoculum. The findings suggested that the bacterial burden at the site of infection depended on the ability to not only form biofilms but also to disperse.

## Discussion

The opportunistic pathogen *P. aeruginosa* causes a variety of human diseases, ranging from acute and chronic persisting infections, fatal bacteremia in immunocompromised patients and superficial skin infections to chronic colonization of patients with CF and chronic obstructive pulmonary disease. This remarkable adaptability depends to a large extent on its ability to transition from a planktonic (unattached) to a biofilm mode of growth and to activate the expression of genes required for viability and growth in a particular environment, while simultaneously repressing genes that are unnecessary or even detrimental for survival in that particular niche. Our findings are in support of previous proteomics and genetic studies of *P. aeruginosa* [1], [2], [3], [8], [9], [77], indicating that planktonic and biofilm cells differ with respect to virulence gene expression with biofilms displaying a biofilm-specific virulence phenotype, and expressing virulence factors up to 30-fold less compared to planktonic cells. The observed down-regulation of virulence factors following biofilm formation likely serves as an additional adaptation for the bacterial communities to avoid recognition and targeting by the immune system. Accordingly, while releasing similar amounts of protein into the supernatant, biofilm cells were found to produce fewer cytotoxic and degradative proteins than planktonic cells (Figs. 1–2). Given that early infection and colonization has been suggested to require the production of a variety of cytotoxic and degradative proteins, some of which function after direct translocation into host cells, the observed difference in cytotoxic and degradative activities is in agreement with planktonic cells causing more considerable host cell damage [1], [78], [79]. In addition to the established acute-to-chronic virulence switch accompanying the motile-to-sessile mode of growth switch, we demonstrate here that dispersion in response to environmental cues is likely another contributing factor to the virulence phenotype of *P. aeruginosa.* However, while our findings support dispersion to contribute to virulence, dispersion is not simply a reversion of the motile-to-sessile switch. Instead, our findings suggest dispersion to represent a distinct virulence phenotype. For one, dispersed cells were characterized by significantly reduced transcript levels of virulence genes compared to both planktonic and biofilms cells. Moreover, while up to 10-fold higher protein concentrations were detected in supernatants of dispersed cells, the cytotoxic and degradative activity levels were found to be intermediary to those detected in supernatants of planktonic and biofilm cells. Our findings of the distinct virulence gene expression profile of dispersed cells are in agreement with our previous findings of biofilm cells in the dispersion stage and dispersed cells exhibiting a phenotype that is distinct from both planktonic and biofilm bacteria [36], [37], [56], [80].

*P. aeruginosa* dispersed cells not only display a virulence phenotype distinct from those of planktonic and biofilm cells, but also likely employ mechanisms enabling them to evade the immune response following the loss of protection associated with biofilm growth. This was apparent in the difference of cytotoxic and degradative enzymes present in supernatants compared to planktonic cells. It is likely that the difference represents a rapid initial response at the level of protein activity to liberate the dispersing cells from the enclosing matrix. While direct evidence of dispersion occurring in the lung environment is lacking, dispersion is widely documented in natural systems and is a basic property of biofilms in general. Moreover, it is likely that ability to degrade the biofilm matrix extends to an ability to degrade the surrounding mucus present in the CF lung, which is chemically similar to known biofilm matrix polymers. Additionally, while *P. aeruginosa* rarely escapes the lung environment, there are indications that dispersion takes place, as dispersed cells are more susceptible to immune function and antimicrobial treatment compared to biofilm cells [47], [56]. For example, Donaldson et al. [81] demonstrated that treatment with aerosolized hypertonic saline causes the vasculature to release water from the blood stream, thereby hydrating the thick mucus, correlated with biofilm bacteria being far more susceptible to the antibiotic amiloride compared to bacteria present in untreated mucus [81]. Differences in virulence factor gene expression may likewise indicate a dispersion-specific adaptation to temporarily avoid the host immune response, enabling dispersed cells to release themselves from the biofilm matrix but remain “immune-masked” until they have either reverted to a planktonic phenotype or have reattached. This was supported by our findings of both Δ*bdlA* and Δ*dipA* mutants, which are impaired in dispersion *in vitro* [45], [46], [56], [58], being attenuated in virulence while demonstrating heightened persistence *in vivo* (Figs. 5–7). The contribution of BdlA and DipA to virulence and persistence furthermore indicated that both proteins likely contribute to dispersion not only *in vitro* but also *in vivo*. The Δ*bdlA* and Δ*dipA* virulence phenotypes further suggested the presence of environmental cues in both the plant and the murine lung environment that contribute to dispersion-inducing conditions. This was apparent as the inability to disperse *in vitro* affected outcomes in both acute and chronic infection models, with dispersion enhancing the virulence of *P. aeruginosa* during an acute infection, but interfering with the ability to establish chronic infections. The manner in which BdlA, DipA, and dispersion contribute to persistence is distinct from biofilm formation, as inactivation of *bdlA* or *dipA* had the opposite effect to overexpression of the phosphodiesterase PA2133, which impairs biofilm formation [76]. Our findings thus likely suggest that in addition to biofilm biomass accumulation, dispersion may also be a contributing factor to *P. aeruginosa* persistence in the murine lung. This was further supported by the finding of a Δ*bdlA* mutant complemented with BdlA harboring the D14A, N23A, W60A, I109A, W182A substitutions, that were previously demonstrated to result in a null phenotype for dispersion, being attenuated in virulence. Additional support for a role of dispersion in *P. aeruginosa* pathogenicity was provided by Δ*dipA* and Δ*rbdA* mutants, that are impaired in dispersion *in vitro* [46], [55], being attenuated in virulence (Figs. 5–6). To our knowledge, this is the first report linking dispersion to virulence of *P. aeruginosa in vivo*.

It is of interest to note that the Δ*bdlA* virulence phenotype is similar to that of a Δ*retS* mutant in the sense that both promote transitions between the planktonic and the sessile lifestyles. While inactivation of *retS* promotes the motile-sessile transition [1], Δ*bdlA* mutants are impaired in the sessile-motile transition, with inactivation of both *retS* or *bdlA* resulting in decreased initial colonization. While the findings suggest that mutations favoring the biofilm lifestyle (by altering either one of the transitions) would be selected for *in vivo*, long-term selection in a host would likely drive populations towards a biofilm phenotype rather than favoring a non-dispersing phenotype. Instead, we expect the dispersion phenotype to be a short-term behavioral change in bacteria and to be associated specifically with acute phase infections.

In summary, we demonstrate for the first time that dispersed cells have a unique virulence phenotype, with dispersion and the reversion to the planktonic mode of growth contributing to virulence and persistence of *P. aeruginosa* in infections. While the ability to disperse *in vitro* in response to various exogenous cues contributes to virulence of *P. aeruginosa* in acute infections, dispersion instead reduces the ability of *P. aeruginosa* to persist at the site of infection. Our observations of dispersion thus reciprocally regulating acute virulence and persistent, chronic infections suggest that dispersion functions as a regulatory switch, mediating the global transition from initial colonization to chronic infections. Thus, our work establishes induced dispersion as not only an integral part of both acute and chronic infections, but also as a potential mechanism of infection control.

## Materials and Methods

### Ethics statement

The use of animals for this study was reviewed by the IACUC committee at the University of Cincinnati (UC) that is a centralized, campus wide animal care and use program under Laboratory Animal Medical Services (LAMS). LAMS is staffed by three full time veterinarians, six veterinary technicians plus animal care staff. One LAMS veterinarian; one veterinary technician and one LAMS husbandry supervisor is on call after hours. LAMS approved the animal care protocol and use protocol/permit/project license. The approved IACUC protocol number is 12-09-06-01 (“*The Molecular Basis of Pseudomonas aeruginosa, Francisella novicida, and Staphylococcus aureus Virulence in Mammalian Hosts*”). All animals were handled in strict accordance with good animal practice and animal keeping. UC has an Animal Welfare Assurance on file with the NIH-OLAW (Assurance Number A-3295-01, expires November 30, 2015). UC fully complies with the Guide for the Care and Use of Laboratory Animals (Guide), the Public Health Service Policy on the Humane Care and Use of Laboratory Animals (PHS Policy) and all U.S. Animal Welfare Act Regulations.

### Bacterial strains, plasmids, media, and culture conditions

*P. aeruginosa* strain PAO1 and its isogenic mutant strain *bdlA* were used in this study. *P. aeruginosa* strain PA14 was used to validate the generality of the findings. All bacterial strains and plasmids used in this study are listed in Table S2. All planktonic strains were grown in Lennox Broth (LB, BD Biosciences) or minimal medium containing glutamate as the sole carbon source [82] in shake flasks at 220 rpm in the absence or presence of 0.1–1.0% arabinose. *Escherichia coli* cultures were grown in LB in the absence or presence of 1 mM Isopropyl β-D-1-thiogalactopyranoside (IPTG). Antibiotics for *P. aeruginosa* were used at the following concentrations: 50–75 µg/mL gentamicin and 200–250 µg/mL carbenicillin.

### Planktonic growth conditions

All planktonic cells used in this study were obtained by reinoculating 0.5 ml of overnight grown cells into 50 ml minimal medium. To obtain exponential phase planktonic cells, bacteria were allowed to grow to an optical density (600 nm) of 0.4. To obtain stationary phase planktonic cells, bacteria were allowed to grow for 8 hr at which time they reached an optical density of 1.2. Under the conditions tested, onset of stationary phase was noted following 6–7 hours of growth. Planktonic cells growth to exponential and stationary phase were furthermore exposed to an additional 18 mM glutamate or the addition of 500 µM sodium nitroprusside (SNP, source of NO [51]) 30 min prior to harvesting the planktonic cells. The timing was chosen to mimic the exposure time of dispersed cells to glutamate and NO.

### Biofilm formation and dispersion

Biofilms were grown in a continuous flow tube reactor system (1 m long size 14 silicone tubing, Masterflex, Cole Parmer, Inc.) at 22°C for up to 5 days to obtain proteins and RNA. For biofilm dispersion assays, biofilms were cultivated in once-through continuous flow tube reactor system composed of size 13 silicone tubing (Masterflex, Cole Parmer, Inc.) at 22°C for 5 days. After 5 days of biofilm growth, biofilm dispersion was induced by the sudden addition of glutamate (18 mM) to the growth medium as previously described [56]. Moreover, dispersion was induced by 500 µM sodium nitroprusside (SNP) which was used as a source of NO [51]. Dispersion was indicated by an increase in turbidity at 600 nm in the effluent from the silicone tubing.

### Analysis of the proteins present in supernatants

Supernatants were obtained from *P. aeruginosa* PAO1 grown planktonically (exponential and stationary phase), as biofilms, and following dispersion in response to NO or changes in the nutrient glutamate concentration. While the volume of the supernatants varied depending on the growth conditions tested, all supernatants tested represented supernatants produced by 1×10^9^ cells. Briefly, supernatants of cells grown planktonically to exponential and stationary phase and as biofilms were collected by centrifugation. Similarly, supernatants of dispersed cells and biofilms remaining attached to the surface upon induction of dispersion (remaining biofilms) were collected after induction of dispersion of 5 day-old *P. aeruginosa* biofilms were collected. The resulting supernatant was filter-sterilized, dialyzed, lyophilized, and subsequently resuspended in sterile water. The concentration of proteins present in supernatants was determined using the Bradford assay (Biorad). Cell pellets were collected and sonicated as previously described [37], and cell debris removed by centrifugation. The protein concentration of the resulting total cell extract was determined using the modified method of Lowry [83]. The resulting protein concentration was then used to calculate the number of cells with 1 ug of total protein being equivalent to 3.6*10^7^ CFU [84].

Proteins present in supernatants were visualized by SDS/PAGE analysis using 12% SDS-gels and Coomassie staining. A total of 10 µg of cell extract was loaded onto the gel. Proteins of interest were excised from the gel, tryptic digested, and subsequently identified by LC-MS/MS essentially as previously described using a QStarXL mass spectrometer (Applied Biosystems) [85].

### Determination of degradative activities present in the extracellular proteome

Supernatants were tested for the presence of hydrolytic enzymes using agar-plate based biochemical assays. To do so, wells having a 7 mm diameter were punched into the agar. A total of 10 µg of secreted proteins resuspended in a total of 100 µl of water was added to per well. The agar plates were subsequently incubated at 37°C for 18 hr before measurements of the halo surrounding the wells were taken. All measurements were normalized by subtracting the diameter of the well. Lipid hydrolysis was determined using Tributyrin HiVeg Agar Base (BD Bioscience) supplemented with 1% tributyrin while proteolysis was determined using milk agar containing 10% skim milk. Hemolysis was determined using trypticase soy agar supplemented with 5% sheep's blood. Psl degradation was determined using 1.5% agar containing 1% Psl. Psl polysaccharide was extracted using the rapid Psl protocol [86]. Each agar plate contained the Psl equivalent obtained from a Psl overexpressing *P. aeruginosa* having an OD of 25. Psl degradation was visualized following 24–48 hr of incubation using 1% iodine solution (Grams iodine, Thermo Scientific). DNAse activity was determined using 100 µg of supernatant protein and a colorimetric assay as previously described by Sinicropi et al. [87] using DNA-methyl green as a substrate. A total of 0.2 mg/ml salmon sperm DNA was used and DNA degradation monitored at 620 nm.

### Quantitative reverse transcriptase PCR (qRT-PCR)

Isolation of mRNA and cDNA synthesis was carried out as previously described [84], [85], [88], [89]. qRT-PCR was performed using the Eppendorf Mastercycler ep *realplex* (Eppendorf AG, Hamburg, Germany) and the KAPA SYBR FAST qPCR Kit (KAPABIOSYSTEMS, Woburn, MA), with oligonucleotides listed in Table S3. *mreB* was used as a control. The stability of *mreB* levels were verified by 16S RNA abundance using primers HDA1/HDA2 [90]. Relative transcript quantitation was accomplished using the ep *realplex* software (Eppendorf AG) by first normalizing transcript abundance (based on Ct value) to *mreB* followed by determining transcript abundance ratios. Melting curve analyses were employed to verify specific single product amplification.

### Virulence testing using *Arabidopsis thaliana*

The role of BdlA in virulence was assessed using the *Arabidopsis thaliana* infection model which provides a quantitative approach and permits the tracking of bacterial cell proliferation *in planta* [91]. Following two weeks of growth in 1/2MS (2.2 g/L Murashige and Skoog basal medium), plants were infected with *P. aeruginosa* PAO1 and its isogenic mutant strains at a final optical density of OD600  = 1.0 in 1/2MS and incubated for a period of 12 days at a 25/22°C, 16 hr-light/8 hr-dark cycle. All plants were inspected daily for signs of infection and/or death as evidenced by wilting, discoloration, and necrosis.

### Model of acute pneumonia infection in CD-1 mice

*P. aeruginosa* strains PAO1 and Δ*bdlA* were grown to late stationary phase (∼ OD 3.0). Cells were harvested, and washed three times with 10 mM MgSO_4_. Bacteria were serial diluted in 10 mM MgSO_4_, mixed in a 1∶1 ratio, and approximately 1×10^7^ cells were intranasally inoculated into the lungs of four CD-1 mice (Charles River, Boston, MA) previously anesthetized with isofluoran. For single infection studies, the mouse lungs were harvested at 24 hr post-infection, homogenized and serially diluted for bacterial burden determination. For competitive index studies, the mouse lungs were harvested at 16 hr post-infection. The bacterial titer is expressed as CFU/g of tissue.

### Chronic lung infection model: Bacterial culture, immobilization of *P. aeruginosa* in agarose beads, and subsequent infection

A chronic infection model established by Cash et al. [72] was used to test whether the dispersion-deficient mutant strains are able to establish a chronic infection. The infection model by Cash was modified by using a murine model instead of a rat model. For the preparation of the agarose beads, *P. aeruginosa* wild type and mutant strain Δ*bdlA*, were grown at 32°C in a low phosphate succinate medium [72] to stationary phase and mixed in a 1/10 ratio with 2% agarose in phosphate-buffered saline (PBS, pH 7.4). The mixture was added to heavy mineral oil equilibrated at 55°C, stirred for 6 min at room temperature, and cooled for 10 min. Free bacteria were removed by washing with 0.5% and 0.25% deoxycholic acid sodium salt in PBS once and then in PBS alone three times. The beads were passively filtered through sterile 200 µm diameter nylon mesh and then verified for size (70- to 150-µm diameter) and uniformity by microscope examination. An aliquot of beads were homogenized, serially diluted, and plated on LB agar plates to enumerate colony-forming units (CFU). A 100 µl inoculum containing 1×10^6^ CFU of viable *P. aeruginosa* entrapped in agarose beads was then introduced into the lungs of adult CD-1 mice (six week old, groups of 10) via the trachea with nonsurgical methods by a 21-gauge blunt-end needle to the back of the tongue above the tracheal opening. Successful delivery of the beads to the lungs was manifested by choking of the mouse immediately after instillation followed by rapid breathing, and was confirmed by harvesting lungs three minutes post-inoculation and CFU determination of lung homogenates. Mice were sacrificed by asphyxiation in a precharged CO_2_ chamber, or by transection of the abdominal aorta after an overdose of pentobarbital (2.0 ml per kg body weight). The bacterial load was expressed as CFU/g of tissue.

### Statistical analysis

A Student's *t*-test was performed for pair-wise comparisons of groups, and multivariant analyses were performed using a 1-Way ANOVA followed by a posteriori test using Sigma Stat software. For the animal studies, Fisher exact test was used for statistical analysis (http://www.quantitativeskills.com/sisa/statistics/fishrhlp.htm).

## Supporting Information

Figure S1 **Dispersion of** ***P. aeruginosa*** **PA14 biofilms correlates with increased release of proteins into the supernatant.** The protein concentration of supernatants was determined using the same number of cells (1e^9^ CFU/ml) regardless of growth conditions. Supernatants were obtained from *P. aeruginosa* PAO1 grown planktonically to exponential and stationary phase, as well as from biofilms and cells dispersed from the biofilm in response to exposure to glutamate (dispersed cells). Experiments were carried out in triplicate. Error bars indicate standard deviation.
(DOCX)

Figure S2 **Detection of degradative activity in the extracellular proteome of** ***P. aeruginosa*** **PA14 is growth-mode dependent.** Degradative activity was determined using 10 µg of supernatant protein in 100 µl of sterile water, followed by measuring the zone of clearance 18 hours post inoculation of the sterile protein solution into the wells of the respective agar plates. Supernatants were obtained from *P. aeruginosa* PA14 grown planktonically to exponential and stationary phase, from biofilms, biofilms post induction of dispersion (remaining biof), and cells dispersed from the biofilm in response to exposure to glutamate (dispersed cells). (A) Proteolytic activity was detected using milk agar plates. (B) Lipid hydrolysis was determined using tributyrine containing agar plates. (C) Hemolytic activity was detected using blood agar plates while (D) Psl degradation was detected on agar plates containing Psl extracted from a *P. aeruginosa* strain overexpressing Psl. Psl degradation was visualized as a zone of clearing following 24 hr incubation and staining the agar plate with iodine. All Experiments were carried out at least in triplicate. Error bars indicate standard deviation.
(DOCX)

Figure S3 **DNA hydrolysis activity of** ***P. aeruginosa*** **PAO1 grown planktonically to exponential and stationary phase, as biofilms, and following dispersion (remaining biofilms, dispersed cells).** A total of 100 µg of supernatant protein was used per spectrophotometric assay. Supernatants were obtained from *P. aeruginosa* PAO1 grown planktonically to exponential and stationary phase as well as from biofilms, biofilms post induction of dispersion (remaining biofilm), and cells dispersed from the biofilm in response to exposure to glutamate (dispersed cells). All experiments were carried out in triplicate. Error bars indicate standard deviation.
(DOCX)

Figure S4 **The virulence phenotype of dispersed cells is distinct from the virulence phenotype of planktonic and biofilm cells.** Differential expression of selected virulence genes by *P. aeruginosa* PA14 grown as biofilms and dispersed cells compared to cells grown planktonically. Differential gene expression was determined by qRT-PCR. Experiments were carried out in triplicate. Error bars indicate standard deviation.
(DOCX)

References S1 **References cited in supplementary material.**
(DOCX)

Table S1 **qRT-PCR analysis.** Transcript levels in *P. aeruginosa* PAO1 biofilm, Δ*bdlA* mutant biofilms, Δ*dipA* mutant biofilms and biofilms overexpressing *bdlA*, as well as dispersed were determined relative to wild-type planktonic cells grown to exponential phase. Biofilms were grown for 5 days under flowing conditions. Dispersion was induced by exposure to glutamate and SNP as previously described [1], [2], [3]. SNP was used as a source of nitric oxide [4]. Experiments were carried out 5 times. Transcript levels of *mreB* were used as control.
(DOCX)

Table S2 **Bacterial strains and plasmids.**
(DOCX)

Table S3 **Primers used.**
(DOCX)
