# Supplementary material for: BdlA, DipA and Induced Dispersion Contribute to Acute Virulence and Chronic Persistence of Pseudomonas aeruginosa
Source: PLoS Pathog. 2014 Jun 5;10(6):e1004168. doi: 10.1371/journal.ppat.1004168 (PMC4047105; doi:10.1371/journal.ppat.1004168)
Supplement: Figure S1 — Dispersion of P. aeruginosa PA14 biofilms correlates with increased release of proteins into the supernatant. The protein concentration of supernatants was determined using the same number of cells (1e9 CFU/ml) regardless of growth conditions. Supernatants were obtained from P. aeruginosa PAO1 grown planktonically to exponential and stationary phase, as well as from biofilms and cells dispersed from the biofilm in response to exposure to glutamate (dispersed cells). Experiments were carried out in triplicate. Error bars indicate standard deviation. (DOCX) [file ppat.1004168.s001.docx]

**Supplementary Figure S1**

**Figure S1. Dispersion of *P. aeruginosa* PA14 biofilms correlates with increased release of proteins into the supernatant.** The protein concentration of supernatants was determined using the same number of cells (1e^9^ CFU/ml) regardless of growth conditions. Supernatants were obtained from *P. aeruginosa* PAO1 grown planktonically to exponential and stationary phase, as well as from biofilms and cells dispersed from the biofilm in response to exposure to glutamate (dispersed cells). Experiments were carried out in triplicate. Error bars indicate standard deviation.
