# Supplementary material for: BdlA, DipA and Induced Dispersion Contribute to Acute Virulence and Chronic Persistence of Pseudomonas aeruginosa
Source: PLoS Pathog. 2014 Jun 5;10(6):e1004168. doi: 10.1371/journal.ppat.1004168 (PMC4047105; doi:10.1371/journal.ppat.1004168)
Supplement: Figure S2 — Detection of degradative activity in the extracellular proteome of P. aeruginosa PA14 is growth-mode dependent. Degradative activity was determined using 10 µg of supernatant protein in 100 µl of sterile water, followed by measuring the zone of clearance 18 hours post inoculation of the sterile protein solution into the wells of the respective agar plates. Supernatants were obtained from P. aeruginosa PA14 grown planktonically to exponential and stationary phase, from biofilms, biofilms post induction of dispersion (remaining biof), and cells dispersed from the biofilm in response to exposure to glutamate (dispersed cells). (A) Proteolytic activity was detected using milk agar plates. (B) Lipid hydrolysis was determined using tributyrine containing agar plates. (C) Hemolytic activity was detected using blood agar plates while (D) Psl degradation was detected on agar plates containing Psl extracted from a P. aeruginosa strain overexpressing Psl. Psl degradation was visualized as a zone of clearing following 24 hr incubation and staining the agar plate with iodine. All Experiments were carried out at least in triplicate. Error bars indicate standard deviation. (DOCX) [file ppat.1004168.s002.docx]

**Supplementary Figure S2**


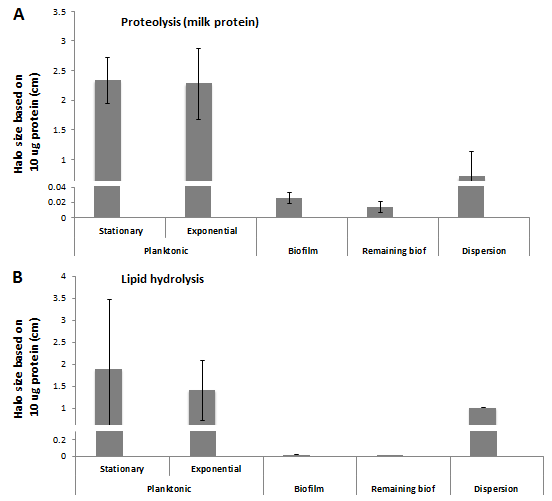


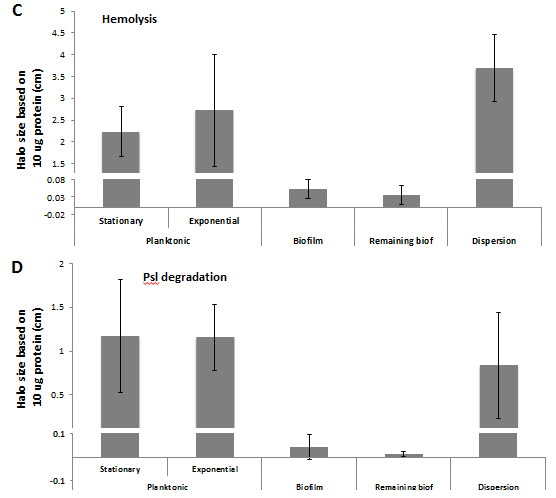


**Figure S2. Detection of degradative activity in the extracellular proteome of *P. aeruginosa* PA14 is growth-mode dependent.** Degradative activity was determined using 10 µg of supernatant protein in 100 µl of sterile water, followed by measuring the zone of clearance 18 hours post inoculation of the sterile protein solution into the wells of the respective agar plates. Supernatants were obtained from *P. aeruginosa* PA14 grown planktonically to exponential and stationary phase, from biofilms, biofilms post induction of dispersion (remaining biof), and cells dispersed from the biofilm in response to exposure to glutamate (dispersed cells). (A) Proteolytic activity was detected using milk agar plates. (B) Lipid hydrolysis was determined using tributyrine containing agar plates. (C) Hemolytic activity was detected using blood agar plates while (D) Psl degradation was detected on agar plates containing Psl extracted from a *P. aeruginosa* strain overexpressing Psl. Psl degradation was visualized as a zone of clearing following 24 hr incubation and staining the agar plate with iodine. All Experiments were carried out at least in triplicate. Error bars indicate standard deviation.
