# Supplementary material for: BdlA, DipA and Induced Dispersion Contribute to Acute Virulence and Chronic Persistence of Pseudomonas aeruginosa
Source: PLoS Pathog. 2014 Jun 5;10(6):e1004168. doi: 10.1371/journal.ppat.1004168 (PMC4047105; doi:10.1371/journal.ppat.1004168)
Supplement: Figure S3 — DNA hydrolysis activity of P. aeruginosa PAO1 grown planktonically to exponential and stationary phase, as biofilms, and following dispersion (remaining biofilms, dispersed cells). A total of 100 µg of supernatant protein was used per spectrophotometric assay. Supernatants were obtained from P. aeruginosa PAO1 grown planktonically to exponential and stationary phase as well as from biofilms, biofilms post induction of dispersion (remaining biofilm), and cells dispersed from the biofilm in response to exposure to glutamate (dispersed cells). All experiments were carried out in triplicate. Error bars indicate standard deviation. (DOCX) [file ppat.1004168.s003.docx]

**Supplementary Figure S3**


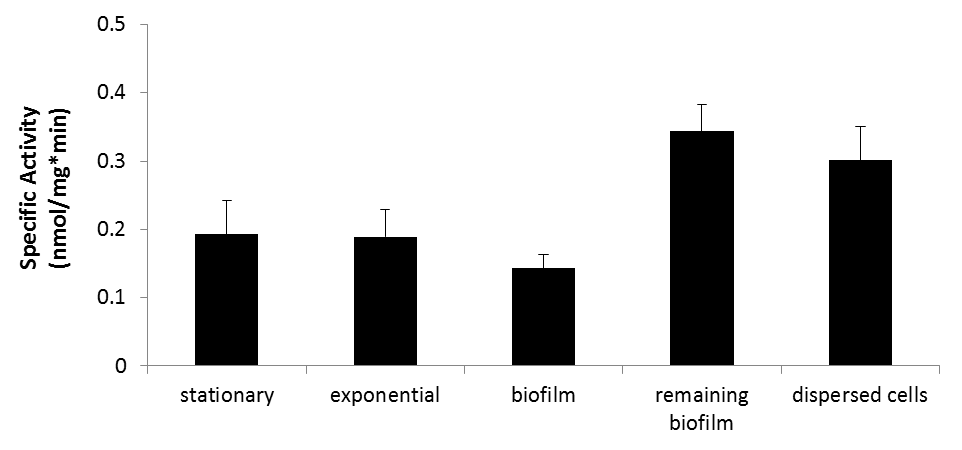


**Planktonic**

**Figure S3. DNA hydrolysis activity of *P. aeruginosa* PAO1 grown planktonically to exponential and stationary phase, as biofilms, and following dispersion (remaining biofilms, dispersed cells).** A total of 100 µg of supernatant protein was used per spectrophotometric assay. Supernatants were obtained from *P. aeruginosa* PAO1 grown planktonically to exponential and stationary phase as well as from biofilms, biofilms post induction of dispersion (remaining biofilm), and cells dispersed from the biofilm in response to exposure to glutamate (dispersed cells). All experiments were carried out in triplicate. Error bars indicate standard deviation.
