# Supplementary material for: BdlA, DipA and Induced Dispersion Contribute to Acute Virulence and Chronic Persistence of Pseudomonas aeruginosa
Source: PLoS Pathog. 2014 Jun 5;10(6):e1004168. doi: 10.1371/journal.ppat.1004168 (PMC4047105; doi:10.1371/journal.ppat.1004168)
Supplement: Figure S4 — The virulence phenotype of dispersed cells is distinct from the virulence phenotype of planktonic and biofilm cells. Differential expression of selected virulence genes by P. aeruginosa PA14 grown as biofilms and dispersed cells compared to cells grown planktonically. Differential gene expression was determined by qRT-PCR. Experiments were carried out in triplicate. Error bars indicate standard deviation. (DOCX) [file ppat.1004168.s004.docx]

**Supplementary Figure S4**

**Figure S4. The virulence phenotype of dispersed cells is distinct from the virulence phenotype of planktonic and biofilm cells.** Differential expression of selected virulence genes by *P. aeruginosa* PA14 grown as biofilms and dispersed cells compared to cells grown planktonically. Differential gene expression was determined by qRT-PCR. Experiments were carried out in triplicate. Error bars indicate standard deviation.
