# Supplementary material for: BdlA, DipA and Induced Dispersion Contribute to Acute Virulence and Chronic Persistence of Pseudomonas aeruginosa
Source: PLoS Pathog. 2014 Jun 5;10(6):e1004168. doi: 10.1371/journal.ppat.1004168 (PMC4047105; doi:10.1371/journal.ppat.1004168)
Supplement: References S1 — References cited in supplementary material. (DOCX) [file ppat.1004168.s005.docx]

**References S1.** References cited in supplementary material.

1. Petrova OE, Sauer K (2012) Dispersion by *Pseudomonas aeruginosa* requires an unusual posttranslational modification of BdlA. Proc National Acad Sci 109 16690-16695.

2. Morgan R, Kohn S, Hwang S-H, Hassett DJ, Sauer K (2006) BdlA, a chemotaxis regulator essential for biofilm dispersion in *Pseudomonas aeruginosa*. J Bacteriol 188: 7335-7343.

3. Basu Roy A, Petrova OE, Sauer K (2012) The phosphodiesterase DipA (PA5017) is essential for *Pseudomonas aeruginosa* biofilm dispersion. J Bacteriol 194: 2904-2915.

4. Barraud N, Hassett DJ, Hwang S-H, Rice SA, Kjelleberg S, et al. (2006) Involvement of nitric oxide in biofilm dispersal of *Pseudomonas aeruginosa*. J Bacteriol 188: 7344-7353.

5. O'Toole GA, Kolter R (1998) Flagellar and twitching motility are necessary for *Pseudomonas aeruginosa* biofilm development. Mol Microbiol 30: 295-304.

6. Jacobs MA, Alwood A, Thaipisuttikul I, Spencer D, Haugen E, et al. (2003) Comprehensive transposon mutant library of *Pseudomonas aeruginosa*. Proc Natl Acad Sci 100: 14339-14344.

7. Ma L, Jackson KD, Landry RM, Parsek MR, Wozniak DJ (2006) Analysis of *Pseudomonas aeruginosa* conditional Psl variants reveals roles for the Psl polysaccharide in adhesion and maintaining biofilm structure postattachment. J Bacteriol 188: 8213-8221.

8. Hickman JW, Tifrea DF, Harwood CS (2005) A chemosensory system that regulates biofilm formation through modulation of cyclic diguanylate levels. Proc Natl Acad Sci 102: 14422-14427.

9. Petrova OE, Sauer K (2012) PAS domain residues and prosthetic group involved in BdlA-dependent dispersion response by *Pseudomonas aeruginosa* biofilms. J Bacteriol 194: 5817-5828.
