# Supplementary material for: BdlA, DipA and Induced Dispersion Contribute to Acute Virulence and Chronic Persistence of Pseudomonas aeruginosa
Source: PLoS Pathog. 2014 Jun 5;10(6):e1004168. doi: 10.1371/journal.ppat.1004168 (PMC4047105; doi:10.1371/journal.ppat.1004168)
Supplement: Table S1 — qRT-PCR analysis. Transcript levels in P. aeruginosa PAO1 biofilm, ΔbdlA mutant biofilms, ΔdipA mutant biofilms and biofilms overexpressing bdlA, as well as dispersed were determined relative to wild-type planktonic cells grown to exponential phase. Biofilms were grown for 5 days under flowing conditions. Dispersion was induced by exposure to glutamate and SNP as previously described [1], [2], [3]. SNP was used as a source of nitric oxide [4]. Experiments were carried out 5 times. Transcript levels of mreB were used as control. (DOCX) [file ppat.1004168.s006.docx]

**Supplementary Table S1**

**Table S1.**  **qRT-PCR analysis.** Transcript levels in *P. aeruginosa* PAO1 biofilm, *ΔbdlA* mutant biofilms, *ΔdipA* mutant biofilms and biofilms overexpressing *bdlA*, as well as dispersed were determined relative to wild-type planktonic cells grown to exponential phase. Biofilms were grown for 5 days under flowing conditions. Dispersion was induced by exposure to glutamate and SNP as previously described [[1](#_ENREF_1),[2](#_ENREF_2),[3](#_ENREF_3)]. SNP was used as a source of nitric oxide [[4](#_ENREF_4)].Experiments were carried out 5 times. Transcript levels of *mreB* were used as control.

| **Strains/Genes** |  | | **Fold change in transcript levels relative to PAO1 grown planktonically** | | | | | |
| --- | --- | --- | --- | --- | --- | --- | --- | --- |
|  | **PAO1 dispersed cells (glutamate)** | **PAO1 dispersed cells (SNP)** | | **PAO1 biofilms** | **PAO1/pJN-*bdlA* biofilms** | **PAO1/pJN-*dipA* biofilms** | ***ΔbdlA* biofilms** | ***ΔbdlA* biofilms** |
| *chiC* | -41.1±0.2* | -90.7±1.0* | | -7.6±0.02* | -120.8±3.0* | -16.3±1.0* | 13.2±0.6* | 4.5±0.2* |
| *hcnA* | -18.3±0.1* | -17.5±0.5* | | -28.0±0.1* | -29.8±0.5* | -16.9±0.1* | 18.9±2.6* | 2.3±0.5 |
| *lasB* | -38.4±0.2* | -51.6±2.1* | | -4.1±0.7* | -30.8±0.01* | -16.5±0.5* | 9.9±1.5* | 4.2±0.2* |
| *pcrV* | -12.8±0.1* | -10.0±0.2* | | 1.55±0.4 | -17.2±0.2* | -1.5±0.1 | -1.7±0.03 | 1.9±0.1 |
| *phzB* | -115.5±0.4* | -55.1±1.3* | | -1.1±0.3 | -13.7±2.0* | -28.2±1.2* | 10.0±1.1* | 2.9±0.1* |
| *pscL* | -7.8±0.2* | -6.3±0.3* | | -4.0±0.07* | -8.7±0.1* | -4.2±0.1* | 1.4±0.4 | 2.0± |
| *rhlA* | -137.7±1.1* | -251.5±5.3* | | -3.7±0.3* | -5.1±0.1* | -46.4±0.1* | 1.6±2.1 | 1.0±0.04 |
| *toxA* | -6.2±0.2* | -7.8±0.2* | | 7.7±0.6* | -2.5±0.1 | -28.4±0.1* | 13.7±3.3* | 3.6±0.4* |

*, significantly different from PAO1 grown planktonically, P-value < 0.01.
