# Supplementary material for: BdlA, DipA and Induced Dispersion Contribute to Acute Virulence and Chronic Persistence of Pseudomonas aeruginosa
Source: PLoS Pathog. 2014 Jun 5;10(6):e1004168. doi: 10.1371/journal.ppat.1004168 (PMC4047105; doi:10.1371/journal.ppat.1004168)
Supplement: Table S2 — Bacterial strains and plasmids. (DOCX) [file ppat.1004168.s007.docx]

**Supplementary Table S2**

**Table S2. Bacterial Strains and Plasmids.**

| **Strains/Plasmids** | | **Relevant genotype or description** | **Source** |
| --- | --- | --- | --- |
| **Strains** | | | |
| ***Escherichia coli*** | | | |
|  | DH5α | F^-^ φ80*lacZ*ΔM15 *Δ(lacZYA-argF)*U169 *rec*A1 *end*A1 *hsd*R17*(r_k_^-^, m_k_^+^) pho*A *sup*E44 *thi-*1 *gyr*A96 *rel*A1 *tonA* | Invitrogen Corp. |
|  | BL21 | F- *ompT* *hsdS*_B_ (r_B_^-^m_B_^-^) *gal dcm* (DE3) | Invitrogen Corp |
| ***P. aeruginosa*** | |  |  |
|  | PA14 | Wild type | [5] |
|  | PAO1 | Wild type | B.H. Holloway |
|  | *ΔbdlA* | *ΔbdlA* in PAO1; Km^R^ | [2] |
|  | *ΔtoxA* | PA1148::ISlacZ in PAO1; Tet^R^ | [6] |
|  | *ΔdipA* | *PA5017*::MAR2xT7; Gm^R^ | [3] |
|  | *ΔrbdA* | *PA0861*::MAR2xT7; Gm^R^ | [3] |
|  | *ΔpcrV* | PA1706::ISlacZ; Tet^R^ | [6] |
|  | *ΔpscL* | PA1725::ISlacZ; Tet^R^ | [6] |
|  | *ΔphzB* | PA1900::ISlacZ; Tet^R^ | [6] |
|  | *ΔhcnA* | PA2139::ISlacZ; Tet^R^ | [6] |
|  | *ΔchiC* | PA2300::ISlacZ; Tet^R^ | [6] |
|  | *ΔrhlA* | PA3479::ISlacZ; Tet^R^ | [6] |
|  | *ΔlasB* | PA3724::ISlacZ; Tet^R^ | [6] |
|  | WFPA801 | Arabinose-inducible expression of *psl* locus in PAO1 | [7] |
| **Plasmids** | | | |
|  | pJN-PA2133 | PA2133 cloned into arabinose-inducible gene expression vector; pBRR-1 MCS; *araC-*P_BAD_; Gm^R^ | [8] |
|  | pJN-*bdlA*-W60A | *bdlA*-V5/His with W60A mutation in pJN105 | [9] |
|  | pJN-*bdlA*-W182A | *bdlA*-V5/His with W182A mutation in pJN105 | [9] |
|  | pJN-*bdlA*-NoPASa | C-terminal V5/6xHis-tagged PASbTar of *bdlA* cloned into pJN105 at *Nhe*I/*Spe*I | [9] |
|  | pUC-*bdlA* | *bdlA* in pUCP20, Carb^R^ | [9] |
|  | pUC-*bdlA-*D14A | *bdlA* with D14A mutation in pUCP20 | [9] |
|  | pUC-*bdlA-*N23A | *bdlA* with N23A mutation in pUCP20 | [9] |
|  | pUC-*bdlA*-E36A | *bdlA* with E36A mutation in pUCP20 | [9] |
|  | pUC-*bdlA*-R75A | *bdlA* with R75A mutation in pUCP20 | [9] |
|  | pUC-*bdlA*-W84A | *bdlA* with W84A mutation in pUCP20 | [9] |
|  | pUC-*bdlA*-I109A | *bdlA* with I109 mutation in pUCP20 | [9] |
