# Supplementary material for: BdlA, DipA and Induced Dispersion Contribute to Acute Virulence and Chronic Persistence of Pseudomonas aeruginosa
Source: PLoS Pathog. 2014 Jun 5;10(6):e1004168. doi: 10.1371/journal.ppat.1004168 (PMC4047105; doi:10.1371/journal.ppat.1004168)
Supplement: Table S3 — Primers used. (DOCX) [file ppat.1004168.s008.docx]

**Supplementary Table S3**

**Table S3. primers used.**

| **Oligonucleotide** | **Sequence** |
| --- | --- |
| chiCf  chiCr  lasBf  lasBr  hcnAf  hcnAr  pcrVf  pcrVr  phzBf  phzBr  pscLf  pscLr  rhlAf  rhlAr  toxAf  toxAr  mreBf  mreBr | CAGTTGCACCAGGCCCGC  GGCCAGGTCGATGGCGC  GCCGCCGACCTGATCG  CAGCACCTGCTCGGCG  CAGACATGACCATCCACCTC  GGTTTCCACCCGCATGCC  CAGCGAGCGGATCGTG  GCGGCGTTGATCTGCG  CCAAAGGCCAGGATCGCCTG  CTGCGGGACCGGAATGCTC  CGACTACCAGGACTAC  CAGGATCACCTGCTTC  GCGCGATGGCGACCAC  CACCACCGAGCTGCGG  CCTCAGCATCACCAGCGAC  GCAGGCGATGACTGATGAC  CTGTCGATCGACCTGGG  CAGCCATCGGCTCTTCG |
